# A novel palladium-2-picolylamine complex grafted on magnetic UiO-66-NH_2_ nanocomposites: as an efficient heterogeneous catalyst for fast Suzuki–Miyaura cross coupling

**DOI:** 10.1039/d5na01048a

**Published:** 2026-02-23

**Authors:** Parastoo Nasri, Masoomeh Norouzi

**Affiliations:** a Department of Chemistry, Faculty of Science, Ilam University P.O. Box 69315516 Ilam Iran m.norozi@ilam.ac.ir

## Abstract

This study reports the facile hydrothermal synthesis of a novel Fe_3_O_4_@UiO-66-NH_2_ magnetic metal–organic framework (MOF) nanocomposite, bypassing the need for additional surface functionalization of Fe_3_O_4_. This amine-functionalized magnetic MOF serves as an efficient and porous support for the covalent immobilization of a palladium-2-picolylamine catalytic complex. The palladium magnetic catalyst exhibits exceptional catalytic activity in Suzuki cross-coupling reactions between diverse aryl halides and phenylboronic acid in high to excellent yields over short reaction times and under environmentally friendly conditions. A key advantage of this catalyst is its facile magnetic recovery and excellent reusability for at least five consecutive cycles with negligible loss in activity. Furthermore, comprehensive heterogeneity testing confirms the robust stability of the catalyst, highlighting its potential for sustainable and efficient catalytic applications.

## Abstract 2

This study reports the facile hydrothermal synthesis of a novel [Fe_3_O_4_@UiO-66-NH(CH_2_CH_2_-2-picolylamine)_2_-Pd(0)] magnetic metal–organic framework (MOF) nanocomposite, bypassing the need for additional surface functionalization of Fe_3_O_4_.

## Graphical abstract

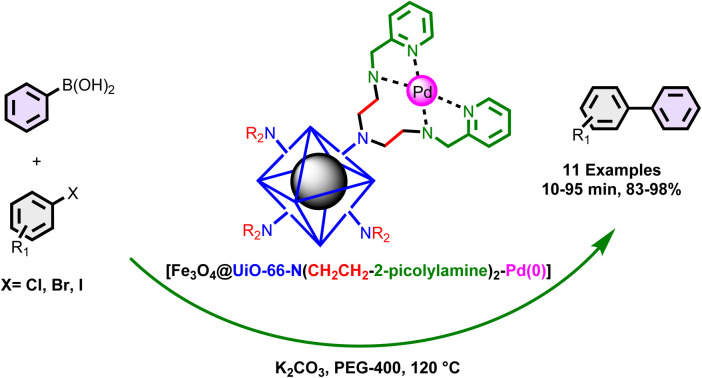

## 1 Introduction

Catalysis is a pivotal tool in modern chemical manufacturing, enabling enhanced reaction rates, improved selectivity, and energy-efficient processes across a vast range of industrial and environmental applications.^1–3^ Catalytic systems are broadly classified into homogeneous, where components are dispersed in the same phase as reactants, and heterogeneous, where components are not in the same phase.^4,5^ Heterogeneous catalysis is particularly favored in industrial applications due to the inherent ease of catalyst separation and recycling.^6–9^ Central to heterogeneous catalytic systems are the supports, which play a critical role in dispersing active sites, modulating their reactivity, and improving catalyst stability and reusability.^10–14^ Conventional supports such as silica,^15^ alumina,^10^ activated carbon,^16^ and natural asphalt,^17^ while extensively used, often lack the structural precision, chemical tunability, and multifunctionality required to meet the growing demands of green and sustainable catalysis.^18^

In this context, metal–organic frameworks (MOFs) have emerged as a transformative class of crystalline, porous materials.^19^ Unlike traditional supports, MOFs can be rationally designed at the molecular level to incorporate catalytic sites within their well-defined cavities or on their surfaces.^20^ As such, MOFs have rapidly advanced from mere gas storage and separation materials to versatile platforms for heterogeneous catalysis.^19,21^ One of the defining advantages of MOFs as catalytic supports lies in their amenability to post-synthetic modification (PSM). Through PSM, preformed MOFs can be functionalized with a wide array of chemical groups, metal complexes, or organocatalysts without compromising their structural integrity.^22–28^ This strategy significantly expands the chemical diversity of MOFs and enables the precise introduction of catalytically active sites.^29–32^

A recent and powerful advancement in this field is the integration of magnetic nanoparticles into MOF structures, yielding magnetic MOFs (MMOFs).^33^ These hybrids retain the functional and structural properties of MOFs while gaining magnetic responsiveness, which enables facile recovery and reuse *via* external magnetic fields. This property is very attractive for practical applications.^33–37^ Among the magnetic MOFs, magnetic UiO-66-NH_2_ (M-UiO-66-NH_2_) has garnered particular interest.^38^ It combines the tunable chemistry and PSM flexibility of UiO-66-NH_2_ with the operational convenience of magnetic separation.^39^ The amino groups in M-UiO-66-NH_2_ facilitate further anchoring of catalytically active species, such as Schiff bases, transition metal ions, or organocatalysts, enabling the creation of site-isolated, multifunctional catalytic systems.^40–43^ Recent developments have highlighted the successful use of M-UiO-66-NH_2_ in a variety of transformations, including multicomponent and coupling reactions.^44–47^ These catalysts exhibit high activity, selectivity, and excellent reusability, underlining the synergetic benefits of MOF architecture, PSM versatility, and magnetic recoverability. Furthermore, their structural robustness allows for repeated use under harsh reaction conditions, making them ideal candidates for sustainable catalytic technologies.^47^

The Suzuki–Miyaura cross-coupling reaction is a pivotal tool in organic synthesis, enabling the efficient formation of carbon–carbon bonds and finding widespread application across pharmaceutical, agrochemical, and materials industries.^48–51^ Its significance stems from its versatility and mild reaction conditions. However, the challenges associated with separating and recycling homogeneous palladium catalysts, typically employed in this reaction, highlight the critical need for efficient heterogeneous alternatives.^17,52,53^ This makes the Suzuki reaction an appropriate benchmark for evaluating the performance and reusability of novel heterogeneous catalysts with immobilized palladium complexes.^54–56^

Although numerous heterogeneous Pd catalysts have been developed for Suzuki–Miyaura cross-coupling reactions, persistent challenges such as limited control over active Pd sites, metal leaching, and poor recyclability continue to hinder practical application.^57,58^ Periodic mesoporous organosilica supports functionalized with ionic liquids have demonstrated efficient and reusable Pd catalysts for aqueous Suzuki reactions, highlighting the benefits of structured porous supports for enhanced activity and recovery.^51^ Early studies using Pd supported on periodic mesoporous organosilicas and magnetic nanoparticles also showed good catalytic performance and recyclability, but these systems rely mostly on immobilization of palladium nanoparticles without precise molecular control of the surrounding environment.^59,60^ More recently, UiO-66-NH_2_ metal–organic frameworks modified with Schiff-base ligands have produced highly active and recyclable Pd-MOF catalysts, emphasizing the ability of MOF platforms to stabilize Pd active species.^59,61,62^ Despite these advances, many reported systems still depend on Pd nanoparticles or simple metal–ligand interactions and often require extensive functionalization.^63^ In contrast, the catalyst described here features covalent immobilization of a well-defined palladium-2-picolylamine complex on a magnetic UiO-66-NH_2_ framework. This design provides strong Pd coordination, facile magnetic recovery without pre-functionalization, excellent catalytic activity under environmentally benign conditions, and robust recyclability, representing a significant advance in catalyst design, stability, and performance for Suzuki–Miyaura coupling.

## 2 Experimental

### 2.1 Materials

All chemicals and solvents used in this study were obtained from Merck Millipore and Sigma-Aldrich and used without further purification.

### 2.2 Synthesis [Fe_3_O_4_@UiO-66-NH(CH_2_CH_2_-2-picolylamine)_2_-Pd(0)] magnetic nanocomposite

At the first step, Fe_3_O_4_ MNPs were synthesized *via* a previously established coprecipitation protocol. Subsequently, Fe_3_O_4_@UiO-66-NH_2_ MMOFs were fabricated through a solvothermal approach. For this purpose, 2.0 g of pre-synthesized Fe_3_O_4_ MNPs were dispersed in 30 mL of *N*,*N*-dimethylformamide (DMF) and subjected to sonication for 30 min to ensure a uniform suspension. Subsequently, 4.0 mmol of zirconium tetrachloride (ZrCl_4_) was introduced, followed by a further 30 min of sonication. To establish an acidic environment, 12 mL of glacial acetic acid was added, and the suspension was sonicated for an additional 30 min. Subsequently, 4.0 mmol of 2-aminoterephthalic acid (H_2_BDC-NH_2_) ligand was incorporated, and the resulting mixture was subjected to 30 min of further sonication. The resultant suspension was then transferred to a 200 mL Teflon-lined stainless-steel autoclave and heated at 160 °C for 72 hours. Following the reaction, the autoclave was allowed to cool to ambient temperature naturally. The synthesized product underwent reflux in a round-bottom flask (RBF) for 24 h to enhance crystallinity and remove residual reactants. After the completion of reaction, the product was isolated by magnetic hot filtration and rigorously purified through successive washes with boiling DMF and ethanol, each followed by magnetic hot filtration to eliminate impurities. Finally, the Fe_3_O_4_@UiO-66-NH_2_ magnetic nanocomposite was dried in an oven at 80 °C for 4 h.

Subsequently, 2.0 g of the Fe_3_O_4_@UiO-66-NH_2_ magnetic nanocomposite were introduced into a RBM equipped with a reflux apparatus, containing 50 mL of DMF and dispersed for 30 minutes to achieve a homogeneous suspension. Then a catalytic amount of sodium iodide and 10 mL of 1,2-dichloroethane were added to the reaction mixture. The reaction was carried out under continuous stirring at 50 °C for 24 hours. The resulting Fe_3_O_4_@UiO-66-NH(CH_2_CH_2_Cl)_2_ nanoparticles were isolated by magnetic separation and subsequently purified through iterative washes with ethanol to remove unreacted precursors. Finally, the obtained nanoparticles were dried under vacuum at 80 °C for 4 hours.

In the next step, 2.0 g of the Fe_3_O_4_@UiO-66-NH(CH_2_CH_2_Cl)_2_ nanocomposite was introduced into an RBF equipped with a reflux apparatus, containing 50 mL of *N*,*N*-dimethylformamide (DMF). The mixture was then sonicated for 30 min to ensure a homogeneous suspension. Then 4.0 mmol of 2-picolylamine were added to the reaction mixture, and the reaction proceeded under continuous stirring at 100 °C for 24 h. Upon completion, the resulting Fe_3_O_4_@UiO-66-NH(CH_2_CH_2_-2-picolylamine)_2_ nanoparticles were isolated by magnetic separation using a neodymium magnet, subsequently purified through repetitive washes with ethanol, and dried under vacuum at 80 °C for 4 hours. Finally, 1.0 g of the Fe_3_O_4_@UiO-66-NH(CH_2_CH_2_-2-picolylamine)_2_ magnetic nanocomposite was dispersed in 50 mL of ethanol and sonicated for 30 min to achieve a homogeneous suspension. Subsequently, 2.0 mmol of palladium(ii) chloride (PdCl_2_) were added to the resulting suspension, and the reaction mixture was stirred under a nitrogen atmosphere at reflux for 24 h. Upon the completion of reaction and cooling to ambient temperature, 4.0 mmol of sodium borohydride (NaBH_4_) were added in portions. The reaction mixture was then stirred under identical conditions for an additional 4 hours resulting in the formation black precipitate. Following this, the resulting black nanoparticles were isolated by magnetic separation and subsequently washed with deionized water and ethanol. The resulting [Fe_3_O_4_@UiO-66-NH(CH_2_CH_2_-2-picolylamine)_2_-Pd(0)] nanocomposite was then dried in an oven at 80 °C for 4 h.

### 2.3 General procedure for Suzuki coupling over the catalysis of [Fe_3_O_4_@UiO-66-NH(CH_2_CH_2_-2-picolylamine)_2_-Pd(0)] magnetic nanocomposite

A RBM flask was charged with a mixture of aryl halide (1 mmol), phenylboronic acid (1.2 mmol), K_2_CO_3_ (3 mmol), 1.3 mol% of the [Fe_3_O_4_@UiO-66-NH(CH_2_CH_2_-2-picolylamine)_2_-Pd(0)] catalyst, and 2 mL of PEG-400 solvent. The reaction was stirred at 120 °C for the required time, as monitored by Thin-layer chromatography (TLC) using *n*-hexane as the eluent. Upon completion, the reaction mixture was diluted with ethyl acetate, and the catalyst was separated using a neodymium magnet. The mixture was then extracted with 25 mL of ethyl acetate and washed with warm water. The organic phase was dried over sodium sulfate, and the solvent was removed by evaporation. The crude product was purified using silica gel as the stationary phase and *n*-hexane as the mobile phase.

## 3 Results and discussion

**Scheme 1:**
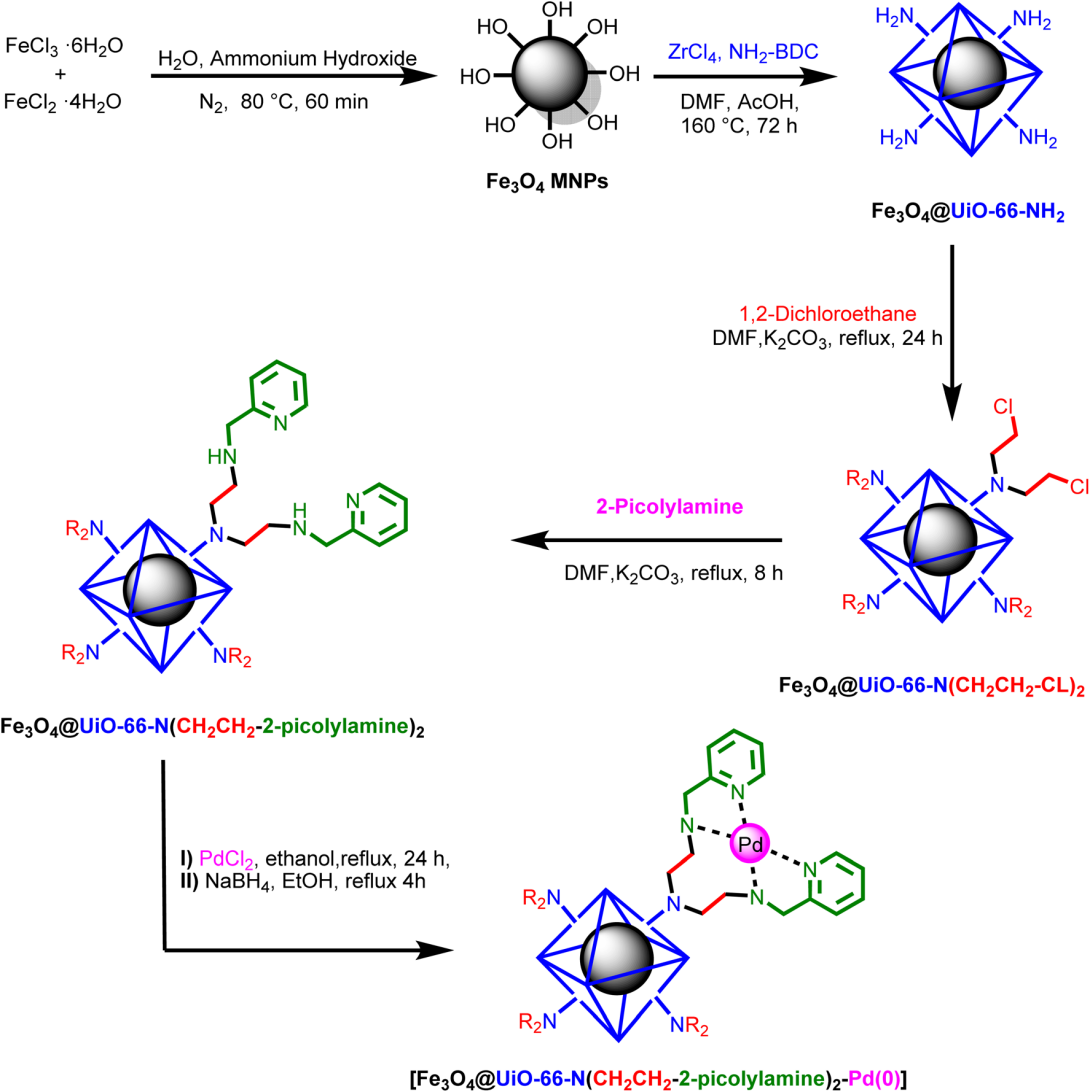
Stepwise synthesis of [Fe_3_O_4_@UiO-66-NH(CH_2_CH_2_-2-picolylamine)_2_-Pd(0)] magnetic nanocomposite.

In this study, a novel palladium (0) complex immobilized on a magnetic metal–organic framework (MOF) was designed and prepared *via* a straightforward four-step procedure (Scheme 1). The process commenced with the solvothermal synthesis of the magnetic MOF, followed by functionalization of its free amine groups through a S_N_2 reaction with 1,2-dichloroethane, thereby introducing electrophilic sites on the magnetic MOF surface. These electrophilic sites were subsequently utilized in a second S_N_2 reaction with 2-picolylamine, leading to the formation of the corresponding heterogenized ligand. The heterogenized ligand was then coordinated to PdCl_2_ to yield the desired palladium (0) complex, which was reduced using NaBH_4_. The resulting magnetic nanocomposite was extensively characterized using a range of techniques, including Fourier-transform infrared spectroscopy (FT-IR), X-ray diffraction (XRD), thermogravimetric analysis–differential scanning calorimetry (TGA-DSC), energy-dispersive X-ray spectroscopy (EDX), inductively coupled plasma optical emission spectrometry (ICP-OES), scanning electron microscopy (SEM), transmission electron microscopy (TEM), nitrogen adsorption–desorption analysis, and vibrating sample magnetometer (VSM) analysis.

### 3.1 Catalyst characterization

**Fig. 1:**
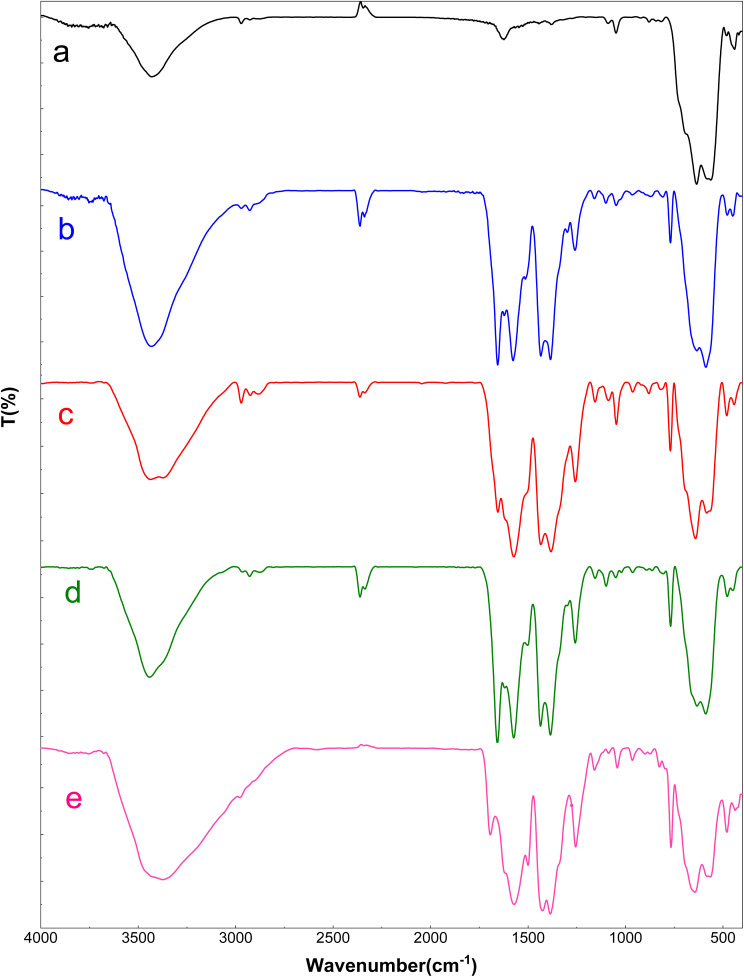
FT-IR spectra of (a) Fe_3_O_4_, (b) Fe_3_O_4_@UiO-66-NH_2_, (c) Fe_3_O_4_@UiO-66-NH(CH_2_CH_2_Cl)_2_, (d) Fe_3_O_4_@UiO-66-NH(CH_2_CH_2_-2-picolylamine)_2_, (e) [Fe_3_O_4_@UiO-66-NH(CH_2_CH_2_-2-picolylamine)_2_-Pd(0)] magnetic nanocomposite.

The FT-IR spectra of Fe_3_O_4_, Fe_3_O_4_@UiO-66-NH_2_, Fe_3_O_4_@UiO-66-NH(CH_2_CH_2_Cl)_2_, Fe_3_O_4_@UiO-66-NH(CH_2_CH_2_-2-picolylamine)_2_ and [Fe_3_O_4_@UiO-66-NH(CH_2_CH_2_-2-picolylamine)_2_-Pd(0)] nanocomposite are illustrated in Fig. 1. The FTIR spectrum of Fe_3_O_4_ shows prominent peaks located at 635 cm^−1^, 563 cm^−1^, and 3430 cm^−1^, which are assigned to Fe–O stretching and hydroxyl (O–H) stretching vibrations, respectively, confirming the formation of iron oxide MNPs (Fig. 1a). The spectrum of Fe_3_O_4_@UiO-66-NH_2_ (Fig. 1b) shows that after the formation of UiO-66-NH_2_ MOF over the Fe_3_O_4_ surface the appearance of new peak at 769 cm^−1^ is due to the O–Zr–O vibration. Moreover, signals at 1655 cm^−1^, 1573 cm^−1^, and 1435 cm^−1^ are attributed to the asymmetric and symmetrical vibrations of the carboxylate moieties in the H_2_BDC-NH_2_ ligand. Additionally, the peak at 1435 cm^−1^ corresponds to the C–C stretching vibration of the aromatic ring. The N–H stretching vibration of the amines is obscured by the overlapping O–H stretching vibrations from the support surface and adsorbed water. Aliphatic CH bands also appear at 2884 cm^−1^, 2924 cm^−1^, and 2971 cm^−1^, confirming the presence of aliphatic C–H moieties in the structure and the formation of the targeted Fe_3_O_4_@UiO-66-NH(CH_2_CH_2_Cl)_2_ structure (Fig. 1c). In the case of Fe_3_O_4_@UiO-66-NH(CH_2_CH_2_-2-picolylamine)_2_, the peaks observed at 3071 cm^−1^, 1637 cm^−1^, and 1436 cm^−1^ are attributed to aromatic C–H stretching, C=N stretching, and aromatic C–C vibrations of the pyridine ring, respectively. These bands overlap with the characteristic peaks of the H2BDC-NH_2_ ligand (Fig. 1d). Finally, as observed in the spectrum of [Fe_3_O_4_@UiO-66-NH(CH_2_CH_2_-2-picolylamine)_2_-Pd(0)], after the addition of palladium, several bands in the 1500–1700 cm^−1^ region broadened and shifted to lower frequencies, which confirming the coordination of nitrogenous sites to the Pd ions and formation of the targeted palladium complex (Fig. 1e).

**Fig. 2:**
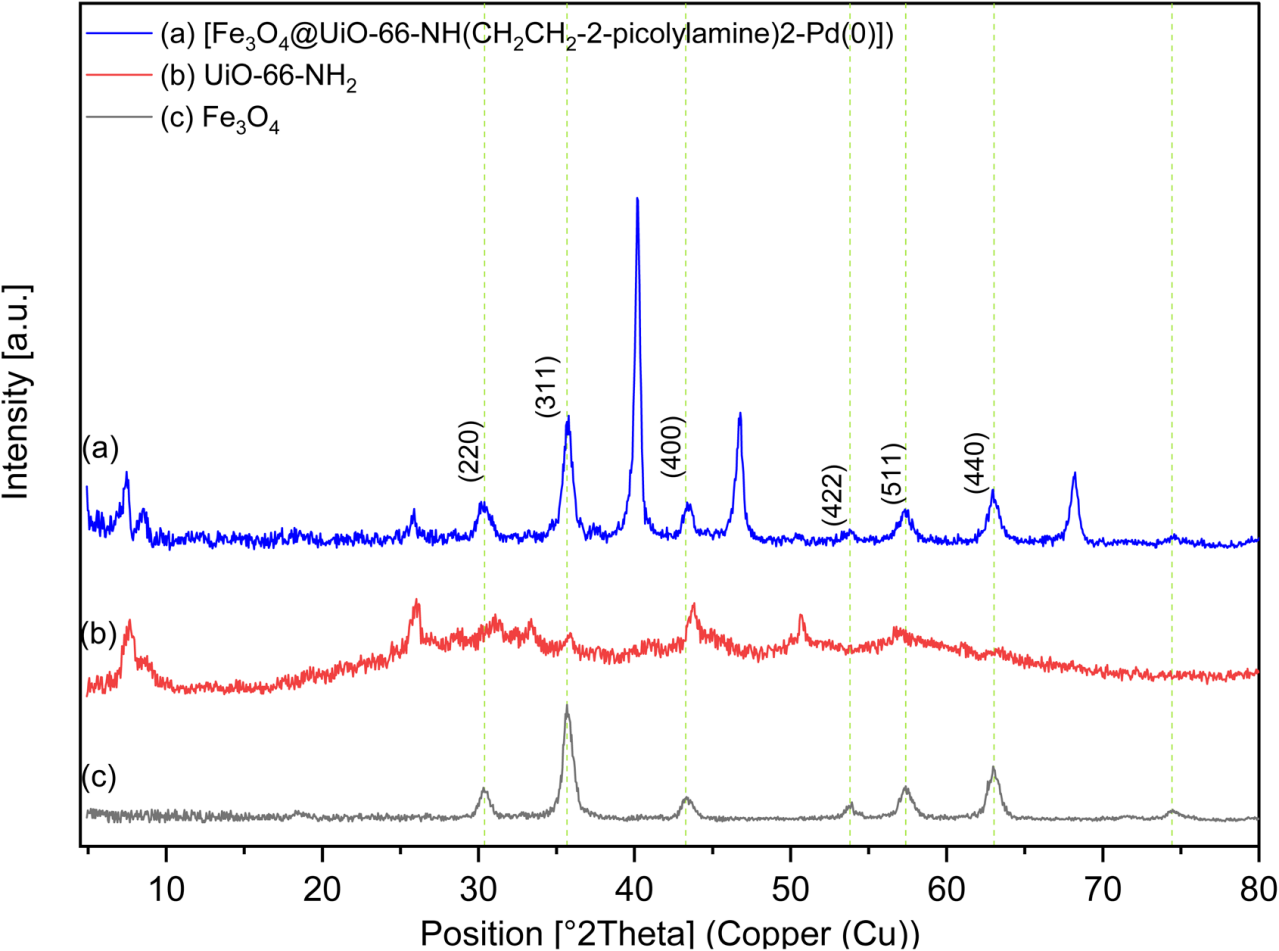
PXRD patterns of (a) Fe_3_O_4_, (b) UiO-66-NH_2_, and (c) [Fe_3_O_4_@UiO-66-NH(CH_2_CH_2_-2-picolylamine)_2_-Pd(0)] magnetic nanocomposite.

The XRD patterns for Fe_3_O_4_, UiO-66-NH_2_, and [Fe_3_O_4_@UiO-66-NH(CH_2_CH_2_-2-picolylamine)_2_-Pd(0)] magnetic nanocomposite are presented in Fig. 2. The XRD pattern of the [Fe_3_O_4_@UiO-66-NH(CH_2_CH_2_-2-picolylamine)_2_-Pd(0)] nanocomposite (Fig. 2a) exhibits characteristic peaks at 2*θ* = 30.27°, 35.72°, 43.47°, 53.75°, 57.33°, and 63.09°, corresponding to the (220), (311), (400), (422), (511), and (440) Miller indices of cubic Fe_3_O_4_ (Fig. 2c) (PCPDFWIN v.2.02, PDF No. 89-0691).^64^ These results confirm that, after several chemical modification steps, the crystallinity of the catalytic support remained intact. Additional peaks observed at 2*θ* = 7.56°, 8.50°, 25.82°, 37.29°, 40.20°, 46.74°, 50.49°, 68.16°and 70.44° correspond to UiO-66-NH_2_ (Zr) (Fig. 2b) MOF and palladium on its surface, further validating the successful synthesis of the targeted catalyst.^17,41^

**Fig. 3:**
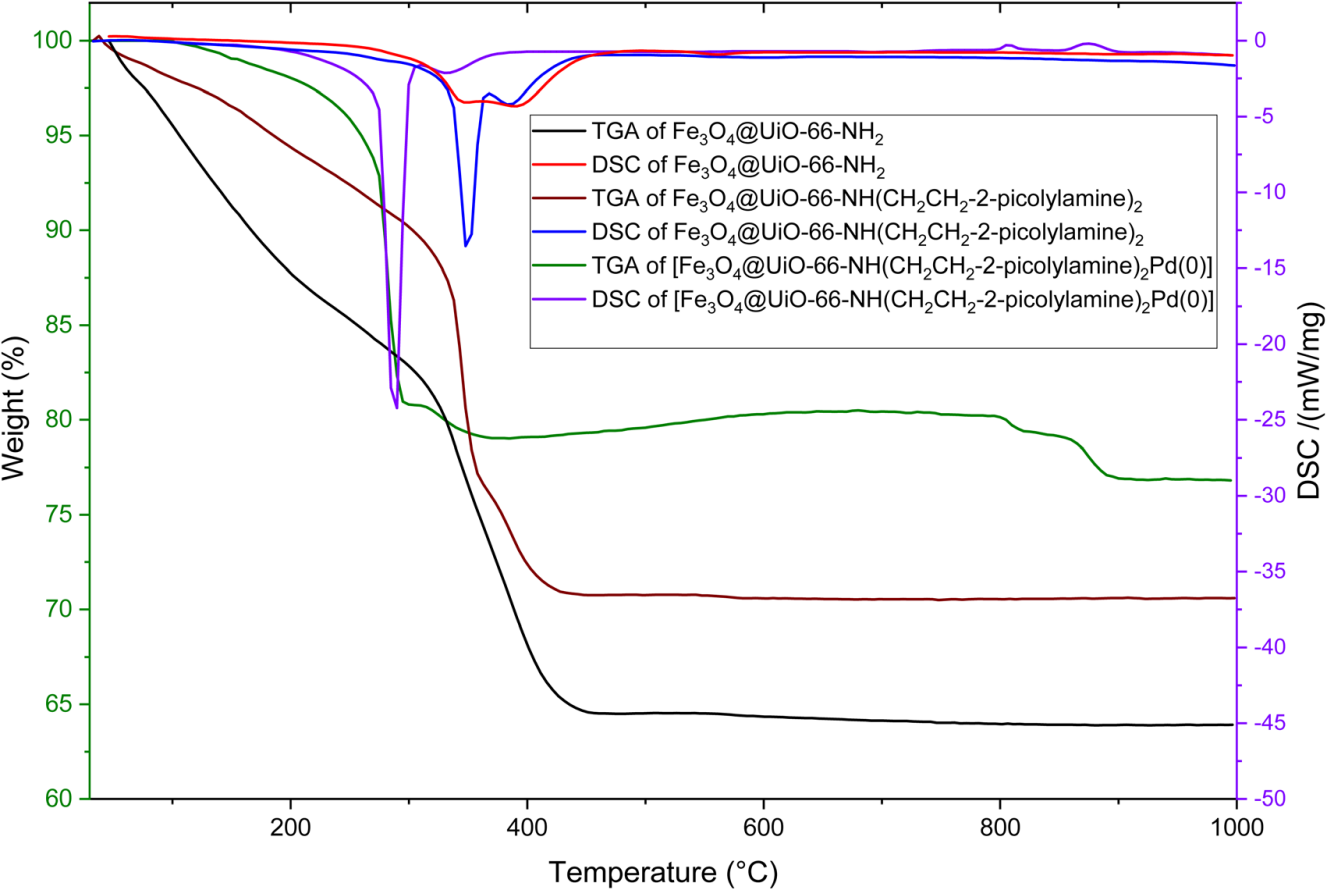
TGA and DSC curves of Fe_3_O_4_@UiO-66-NH_2_, Fe_3_O_4_@UiO-66-NH(CH_2_CH_2_-2-picolylamine)_2_ and [Fe_3_O_4_@UiO-66-NH(CH_2_CH_2_-2-picolylamine)_2_-Pd(0)] magnetic nanocomposite.

The thermal properties of the Fe_3_O_4_@UiO-66-NH_2_, Fe_3_O_4_@UiO-66-NH(CH_2_CH_2_-2-picolylamine)_2_, and [Fe_3_O_4_@UiO-66-NH(CH_2_CH_2_-2-picolylamine)_2_-Pd(0)] nanocomposites were analyzed using TGA and DSC (Fig. 3). The TGA curves indicate that all samples exhibit an initial weight loss below 200 °C, which is attributed to the evaporation of moisture and adsorbed water within the pores of these nanocomposites. Notably, Fe_3_O_4_@UiO-66-NH_2_ and Fe_3_O_4_@UiO-66-NH(CH_2_CH_2_-2-picolylamine)_2_ show the highest weight losses (approximately 13% and 6%, respectively) within this temperature range. In contrast, the [Fe_3_O_4_@UiO-66-NH(CH_2_CH_2_-2-picolylamine)_2_-Pd(0)] magnetic nanocomposite experiences a much smaller loss of only about 2%, confirming that the catalyst sample is well dried.

The Fe_3_O_4_@UiO-66-NH_2_, Fe_3_O_4_@UiO-66-NH(CH_2_CH_2_-2-picolylamine)_2_ and [Fe_3_O_4_@UiO-66-NH(CH_2_CH_2_-2-picolylamine)_2_-Pd(0)] samples exhibit their main weight losses of approximately 23%, 24%, and 19%, respectively, in the temperature range of 200–600 °C. This loss is attributed to the pyrolysis of the organic content on the surface of the magnetic nanocomposite particles (MNPS). The presence of 23% hydrocarbon in Fe_3_O_4_@UiO-66-NH_2_ confirms the presence of H2BDC-NH_2_ and the formation of the targeted magnetic MOF. Additionally, the introduction of the ligand leads to an increases in carbon content. However, when palladium was added to the nanocomposite, the weight loss decreased by about 5%. This reduction is due to the inorganic nature of palladium, which does not participate in the pyrolysis reaction (burning process). Consequently, the chemical composition of the sample is altered by the addition of palladium, resulting in a lower weight loss compared to its Fe_3_O_4_@UiO-66-NH(CH_2_CH_2_-2-picolylamine)_2_ precursor. The comparison of DSC curves reveals that the addition of CH_2_CH_2_-2-picolylamine causes the initial broad peak in Fe_3_O_4_@UiO-66-NH_2_ to split into two separate peaks in Fe_3_O_4_@UiO-66-NH(CH_2_CH_2_-2-picolylamine)_2_, indicating stepwise pyrolysis and the presence of new components in the sample. Moreover, the introduction of palladium shifts these peaks to lower temperatures, illustrating the effect of Pd on the pyrolysis reaction. These observations confirm the successful formation of the targeted catalyst and demonstrate its thermal stability up to 200 °C under atmospheric conditions.

**Fig. 4:**
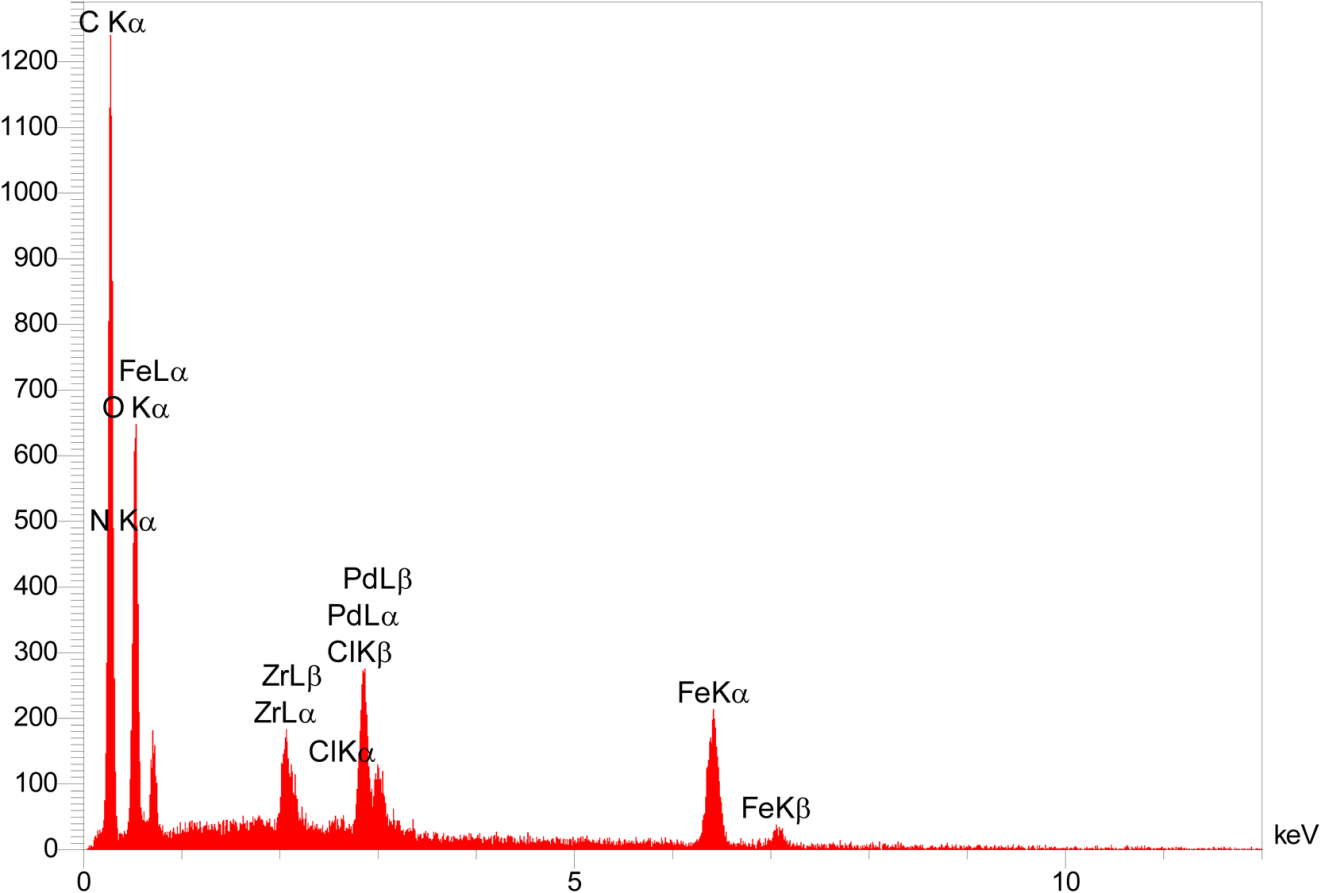
EDX analysis of [Fe_3_O_4_@UiO-66-NH(CH_2_CH_2_-2-picolylamine)_2_-Pd(0)].

**Fig. 5:**
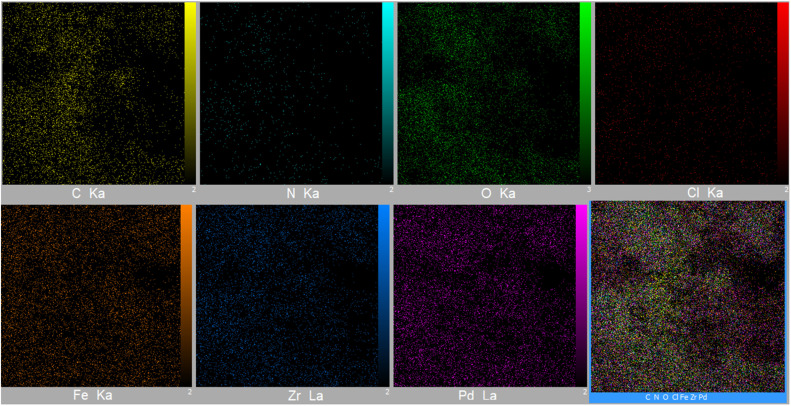
Elemental mapping images of [Fe_3_O_4_@UiO-66-NH(CH_2_CH_2_-2-picolylamine)_2_-Pd(0)].

EDS analysis of the composite material confirmed the presence of iron and oxygen, which is consistent with the expected elemental composition of the Fe_3_O_4_ core. The detection of zirconium, carbon, and nitrogen provides evidence for the successful formation of the UiO-66-NH_2_ MOF shell. Furthermore, the presence of palladium confirms the successful incorporation of the active catalytic sites, indicating the completion of the catalyst synthesis (Fig. 4). ICP-OES was employed to quantify the palladium loading in the catalyst, yielding a value of 0.212 × 10^−2^ mol g^−1^. Additionally, elemental mapping *via* EDS revealed a homogeneous distribution of palladium throughout the composite particles, although a lower surface concentration was observed relative to carbon. The presence of nitrogen atoms within the MOF structure, acting as potential electron-donating ligands, further supports the proposed coordination environment of the incorporated palladium species (Fig. 5).

**Fig. 6:**
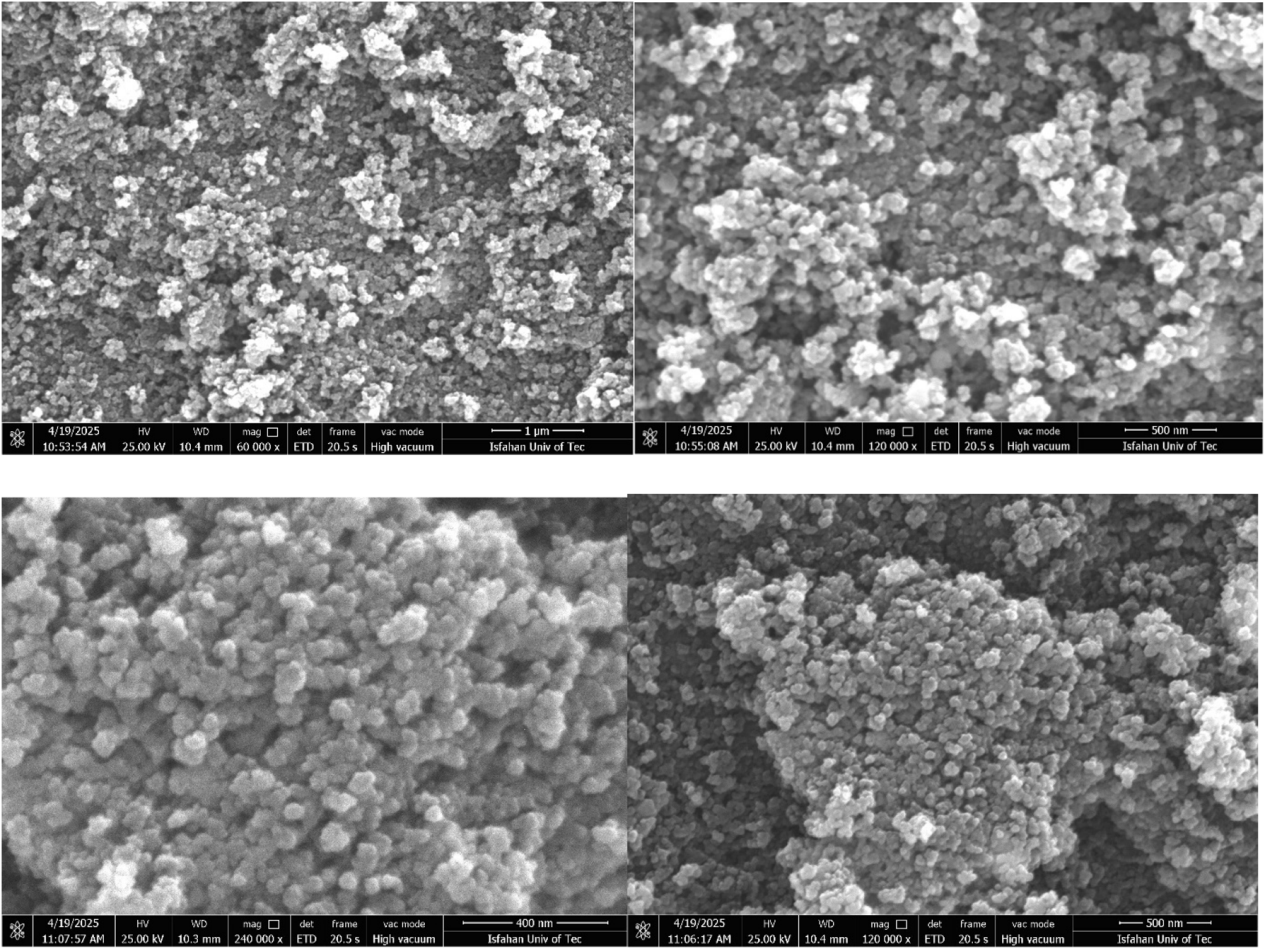
SEM images of [Fe_3_O_4_@UiO-66-NH(CH_2_CH_2_-2-picolylamine)_2_-Pd(0)].

SEM images revealed that the [Fe_3_O_4_@UiO-66-NH(CH_2_CH_2_-2-picolylamine)_2_-Pd(0)] particles predominantly exhibit a roughly spherical or granular morphology (Fig. 6). These primary particles, estimated to be in the nanometer size range, tend to agglomerate, forming irregular clusters and consequently generating a porous structure at the macroscopic level. The observed agglomeration and resulting porosity could significantly influence the material's surface area and accessibility of active sites, potentially impacting its catalytic performance. The surface texture of the individual particles appears somewhat rough, which might further contribute to the overall surface area.

**Fig. 7:**
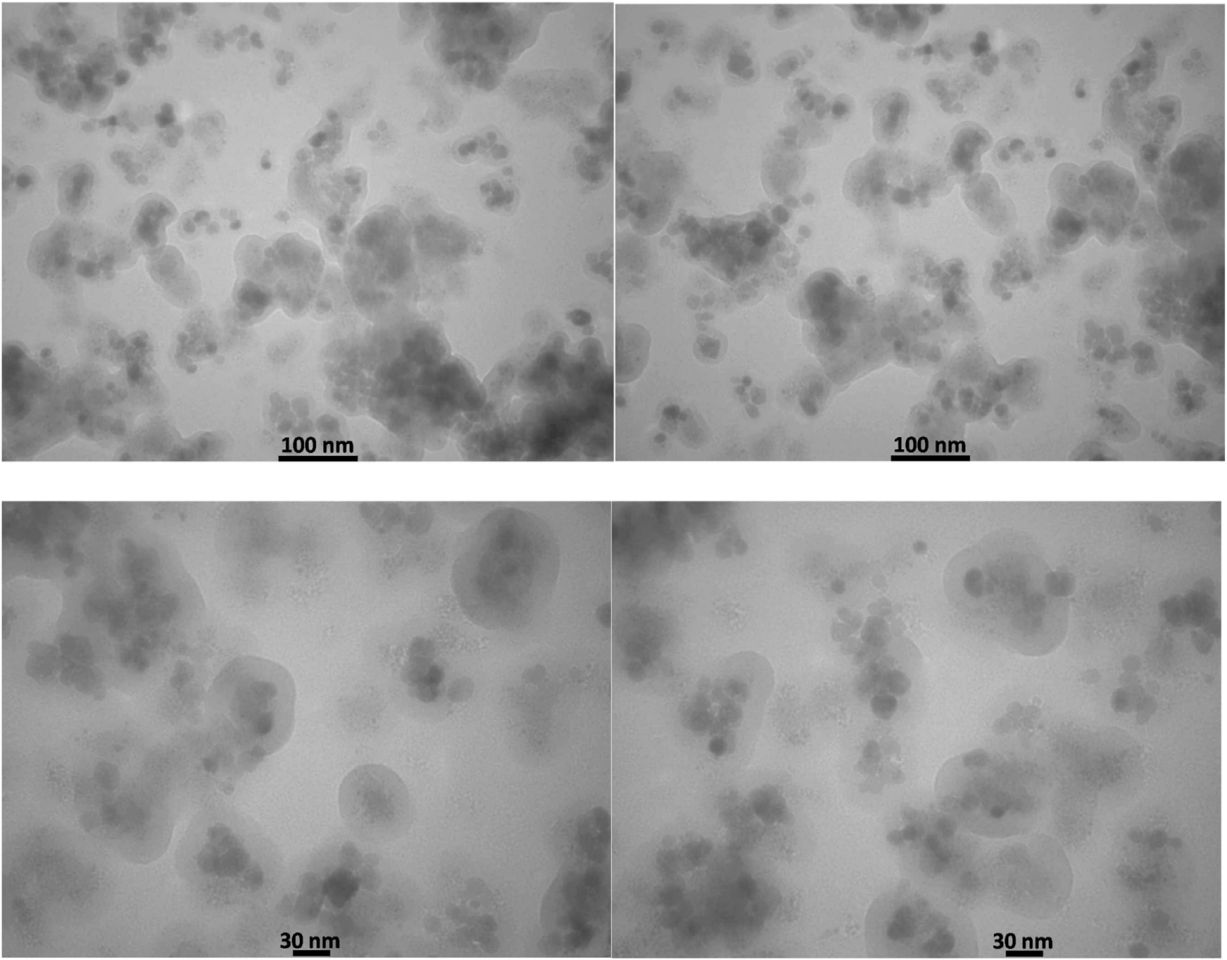
TEM images of [Fe_3_O_4_@UiO-66-NH(CH_2_CH_2_-2-picolylamine)_2_-Pd(0)].

TEM analysis provided compelling evidence for the formation of the [Fe_3_O_4_@UiO-66-NH(CH_2_CH_2_-2-picolylamine)_2_-Pd(0)] nanocomposite, showcasing a well-defined core–shell structure (Fig. 7). The images illustrate particles with an average diameter of 25 nm, characterized by a highly porous UiO-66-NH_2_ shell surrounding the magnetic Fe_3_O_4_ core. The observed porous morphology of the shell is consistent with the expected characteristics of the MOF component and confirms the successful fabrication of the desired catalyst.

**Fig. 8:**
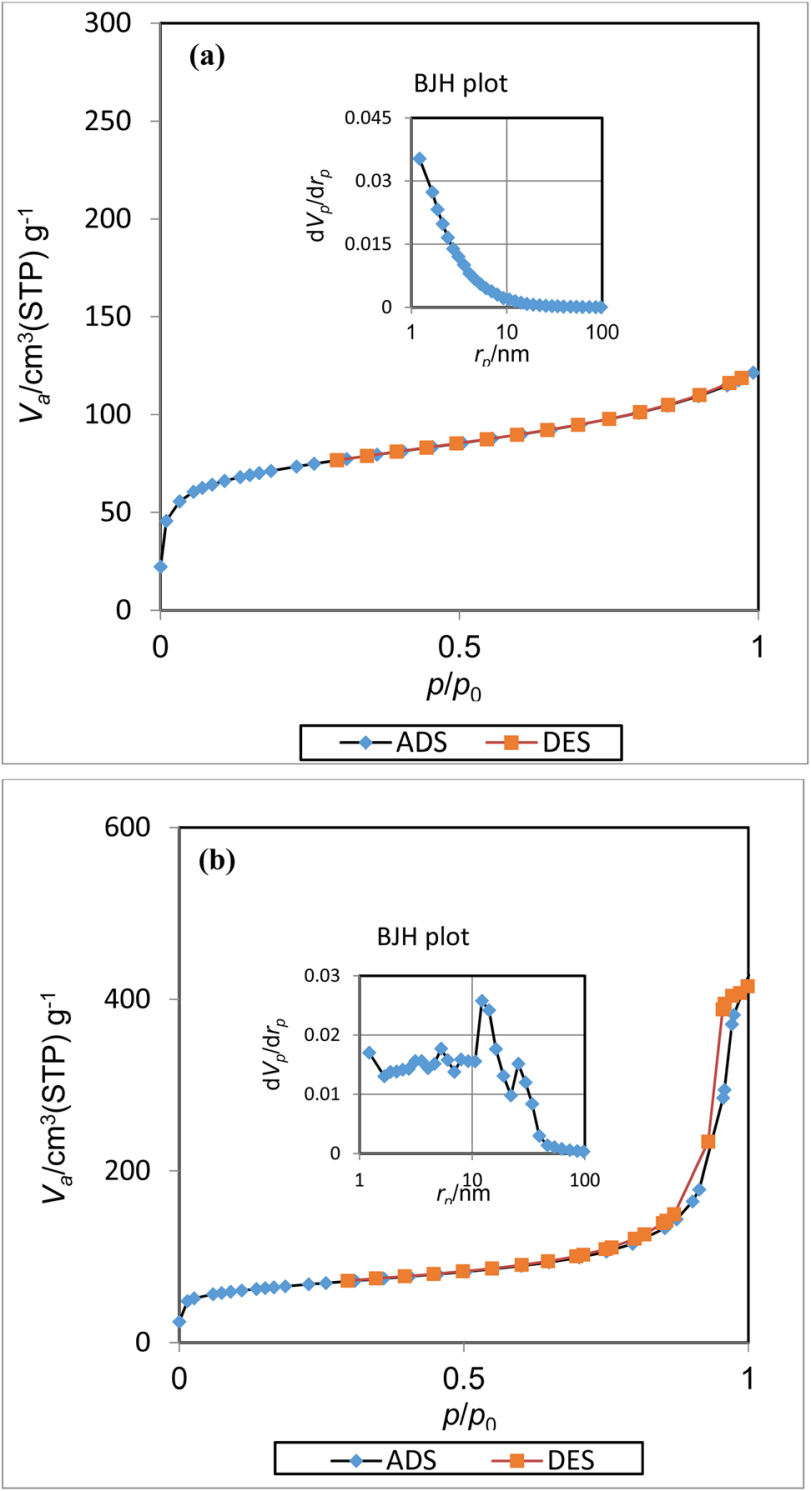
Nitrogen adsorption–desorption isotherms and BJH plot of (a) Fe_3_O_4_@UiO-66-NH_2_ and (b) [Fe_3_O_4_@UiO-66-NH(CH_2_CH_2_-2-picolylamine)_2_-Pd(0)] magnetic.

The nitrogen adsorption–desorption curves and pore size distributions of Fe_3_O_4_@UiO-66-NH_2_ and [Fe_3_O_4_@UiO-66-NH(CH_2_CH_2_-2-picolylamine)_2_-Pd(0)] are demonstrated in Fig. 8. Noticeably, Fe_3_O_4_@UiO-66-NH_2_ exhibits a type I isotherm and significant microporous adsorption is reported at very low relative pressures (Fig. 8a). In contrast, the isotherm of [Fe_3_O_4_@UiO-66-NH(CH_2_CH_2_-2-picolylamine)_2_-Pd(0)] (Fig. 8b) is of type IV with a hysteresis loop H3. These isotherms confirm a hierarchical porous structure consisting of intrinsic MOF microporous pores and secondary mesopores. Pore size distribution curves using the BJH method can also confirm the mesoporous structure.

The functionalization of [(CH_2_CH_2_-2-picolylamine)2-Pd(0)] caused a decrease in the surface area from 258.38 m^2^ g^−1^ to 235.36 m^2^ g^−1^ in the nanocatalyst compared to Fe_3_O_4_@UiO-66-NH_2_, which is attributed to the blocking or occupation of some pores by attached organic moieties. It is worth noting that the average pore diameter increased significantly from about 2.90 nm to ∼10.76 nm and the total pore volume increased to 0.6331 cm^3^ g^−1^at *p*/*p*_0_ = 0.990. This trend is accompanied by a significant increase in the mesoporous space and the formation of a more porous network.

**Fig. 9:**
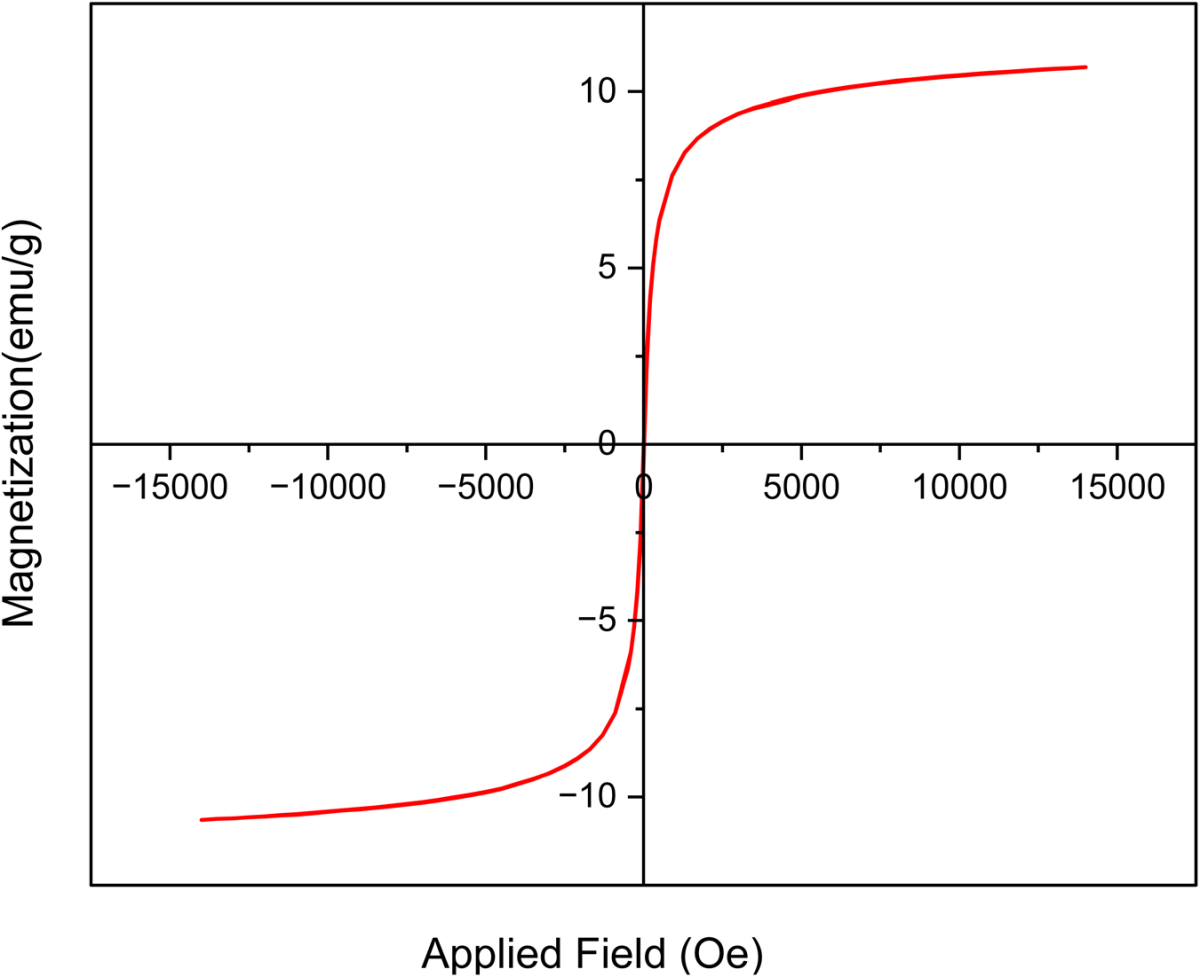
The VSM analysis of [Fe_3_O_4_@UiO-66-NH(CH_2_CH_2_-2-picolylamine)_2_-Pd(0)] magnetic nanocomposite.

The magnetic properties of the [Fe_3_O_4_@UiO-66-NH(CH_2_CH_2_-2-picolylamine)_2_-Pd(0)] catalyst were evaluated using VSM analysis. Fig. 9 shows that the prepared catalyst exhibits a saturation magnetization (MS) value of 10.68 emu per g, which is lower than the MS value of bare Fe_3_O_4_ reported in the literature. This decrease in magnetization can be attributed to the coating of the magnetic nanoparticles with diamagnetic ligands and metals, which reduces the ferrimagnetic iron content in the sample. Consequently, these results confirm the successful surface functionalization. Despite, the MS value remains sufficiently high to allow for the effective separation of the catalyst from the reaction medium using a neodymium magnet.

### 3.2 Catalytic study

Following the structural characterization of the [Fe_3_O_4_@UiO-66-NH(CH_2_CH_2_-2-picolylamine)_2_-Pd(0)] magnetic nanocomposite, its catalytic performance was evaluated using the Suzuki cross-coupling reaction as a representative model for C–C bond formation. To establish optimal conditions, the coupling of iodobenzene with phenylboronic acid was selected as the model reaction. Subsequently, the influence of various reaction parameters, including catalyst loading, base, solvent, and temperature, was systematically investigated. Initial optimization experiments were conducted to ascertain the necessity of the catalyst for the Suzuki–Miyaura cross-coupling reaction. Under catalyst-free conditions, even after an extended reaction time of 24 h, no TLC detectable product formation was observed, confirming the absence of background reactivity (Table 1, entry 1). Furthermore, individual components such as Fe_3_O_4_, Fe_3_O_4_@UiO-66-NH_2_, and Fe_3_O_4_@UiO-66-NH(CH_2_CH_2_-2-picolylamine)_2_ were also tested under identical conditions and found to be catalytically inactive. In stark contrast, the introduction of the [Fe_3_O_4_@UiO-66-NH(CH_2_CH_2_-2-picolylamine)_2_-Pd(0)] nanocatalyst facilitated the reaction efficiently, leading to the successful formation of the desired biaryl product. This apparent difference unequivocally underscores the indispensable catalytic activity of the palladium species immobilized within the nanocomposite in promoting this C–C bond-forming transformation. The amount of catalyst was found to play a critical role in optimizing the reaction efficiency. Among the various loadings tested, 1.3 mol% of the [Fe_3_O_4_@UiO-66-NH(CH_2_CH_2_-2-picolylamine)_2_-Pd(0)] nanocatalyst provided the highest yield of 98% (Table 1, Entry 8). The influence of solvent type was also investigated using green solvents such as water, ethanol, and polyethylene glycol (PEG-400). Among these, PEG-400 gave superior results, likely due to its higher boiling point, phase-transfer capability, and potential reducing effect, which may support the catalytic cycle. At the next step, various bases, including K_2_CO_3_, Na_2_CO_3_, NaOH, NaOAc, and KOtBu, were evaluated to determine their influence on the reaction outcome. Among them, K_2_CO_3_ consistently afforded the highest yields, confirming its suitability as the optimal base for this transformation. Finally, temperature optimization revealed that the reaction did not proceed at room temperature, confirming the necessity of thermal activation. Therefore, 120 °C was identified as the optimal temperature. Based on the optimization studies, the optimal reaction conditions were determined to be 1.3 mol% of catalyst, K_2_CO_3_ as the base, in PEG-400 at 120 °C.

**Table 1:** Optimization of Suzuki C–C coupling reaction over the catalysis of [Fe_3_O_4_@UiO-66-NH(CH_2_CH_2_-2-picolylamine)_2_-Pd(0)] catalyst

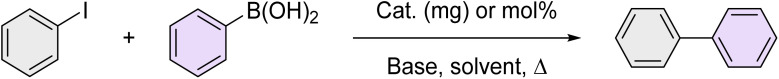							
Entry	Catalyst	Catalyst amount (mol%)	Base	Solvent	Temp. (°C)	Time (min)	Yield (%)^a^^,^^b^
1	–	–	K_2_CO_3_	PEG-400	120	1 day	NR
2	Fe_3_O_4_	5 mg	K_2_CO_3_	PEG-400	120	5h	NR
3	Fe_3_O_4_@UiO-66-NH_2_	5 mg	K_2_CO_3_	PEG-400	120	5h	NR
4	Fe_3_O_4_@UiO-66-NH(CH_2_CH_2_-2-picolylamine)_2_	5 mg	K_2_CO_3_	PEG-400	120	5h	NR
5	[Fe_3_O_4_@UiO-66-NH(CH_2_CH_2_-2-picolylamine)_2_-Pd(0)]	0.4	K_2_CO_3_	PEG-400	120	5h	48
6	[Fe_3_O_4_@UiO-66-NH(CH_2_CH_2_-2-picolylamine)_2_-Pd(0)]	0.8	K_2_CO_3_	PEG-400	120	15	87
7	[Fe_3_O_4_@UiO-66-NH(CH_2_CH_2_-2-picolylamine)_2_-Pd(0)]	1	K_2_CO_3_	PEG-400	120	15	94
8	[Fe_3_O_4_@UiO-66-NH(CH_2_CH_2_-2-picolylamine)_2_-Pd(0)]	1.3	K_2_CO_3_	PEG-400	120	15	98
9	[Fe_3_O_4_@UiO-66-NH(CH_2_CH_2_-2-picolylamine)_2_-Pd(0)]	1.5	K_2_CO_3_	PEG-400	120	15	98
10	[Fe_3_O_4_@UiO-66-NH(CH_2_CH_2_-2-picolylamine)_2_-Pd(0)]	1.3	K_2_CO_3_	Water	Reflux	15	63
11	[Fe_3_O_4_@UiO-66-NH(CH_2_CH_2_-2-picolylamine)_2_-Pd(0)]	1.3	K_2_CO_3_	EtOH	Reflux	15	78
12	[Fe_3_O_4_@UiO-66-NH(CH_2_CH_2_-2-picolylamine)_2_-Pd(0)]	1.3	Na_2_CO_3_	PEG-400	120	15	91
13	[Fe_3_O_4_@UiO-66-NH(CH_2_CH_2_-2-picolylamine)_2_-Pd(0)]	1.3	NaOH	EtOH	120	15	83
14	[Fe_3_O_4_@UiO-66-NH(CH_2_CH_2_-2-picolylamine)_2_-Pd(0)]	1.3	NaOAC	PEG-400	120	15	63
15	[Fe_3_O_4_@UiO-66-NH(CH_2_CH_2_-2-picolylamine)_2_-Pd(0)]	1.3	KOtBu	PEG-400	120	15	65
16	[Fe_3_O_4_@UiO-66-NH(CH_2_CH_2_-2-picolylamine)_2_-Pd(0)]	1.3	K_2_CO_3_	PEG-400	100	15	87
17	[Fe_3_O_4_@UiO-66-NH(CH_2_CH_2_-2-picolylamine)_2_-Pd(0)]	1.3	K_2_CO_3_	PEG-400	r.t	120	Trace

a Isolated yield.

b Conditions: iodobenzene (1 mmol), phenylboronic acid (1.2 mmol), base (3 mmol), catalyst (mg mol^−1^%) and solvent (2 mL).

To further validate the developed protocol's efficiency and broad scope, we examined a diverse set of aryl halides. These varied in both halogen type (iodine, bromine, and chlorine) and the electronic nature of substituents on the phenyl ring. As anticipated, aryl iodides consistently exhibited the highest reactivity, followed by aryl bromides, with aryl chlorides being the least reactive. This trend is fully consistent their known bond dissociation energies. Additionally, substrates bearing electron-withdrawing groups demonstrated enhanced reactivity, affording the desired products in shorter reaction times and with higher yields compared to those bearing electron-donating groups. To assess the chemoselectivity of this methodology, 1-chloro-4-iodobenzene was subjected to the reaction with an equimolar amount of phenylating agent. The coupling occurred selectively at the more reactive C–I position, exclusively yielding the corresponding para-chlorinated biphenyl derivative with no further transformation observed at the C–Cl site. This outcome unequivocally confirms the high selectivity of this method toward more reactive aryl halides (Table 2).

**Table 2:** The substrate scope of Suzuki reaction over the catalysis of [Fe_3_O_4_@UiO-66-NH(CH_2_CH_2_-2-picolylamine)_2_-Pd(0)] complex

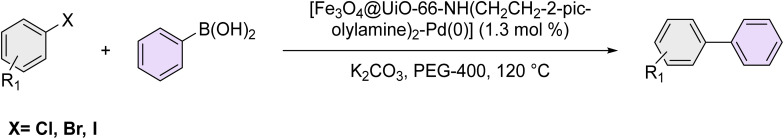						
Entry	Aryl halide	Product	Time (min)	Yield (%)^a^^,^^b^	Melting point (°C)	
					Measured	Literature (ref.)
1	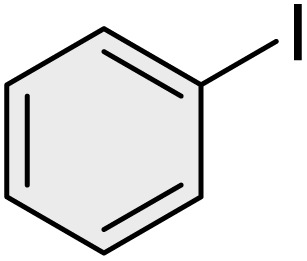	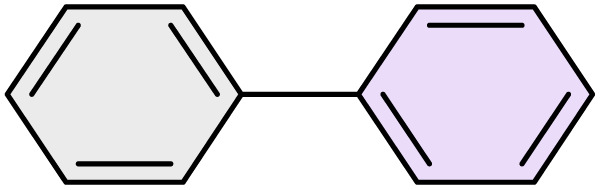	15	98	61–63	62–64 (ref. 65)
2	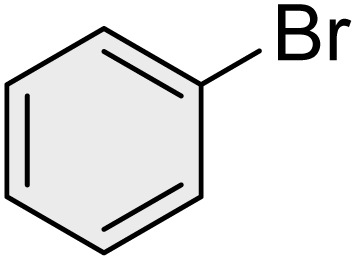	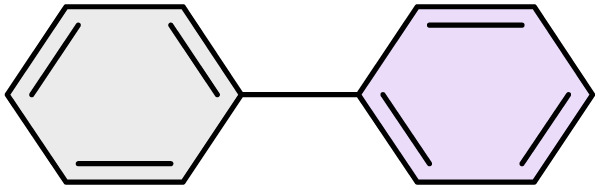	25	95	62–63	62–64 (ref. 65)
3	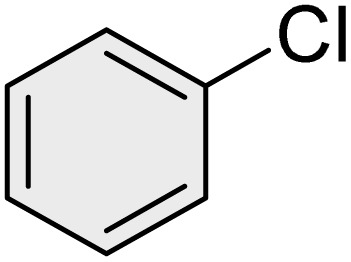	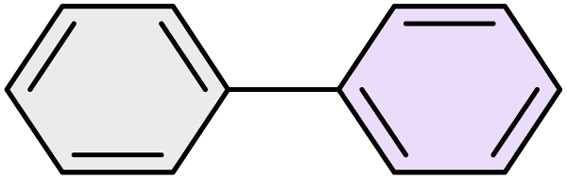	95	83	62–64	62–64 (ref. 65)
4	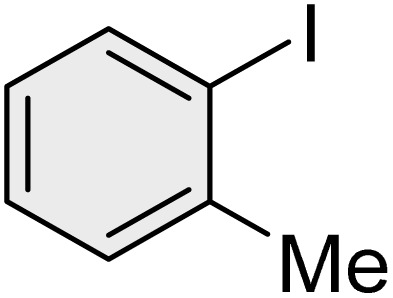	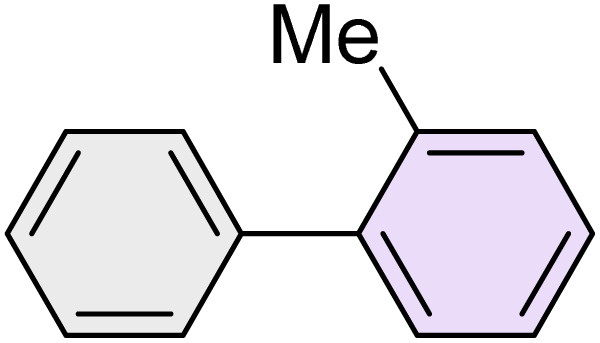	25	94	Oil	Oil (ref. 66)
5	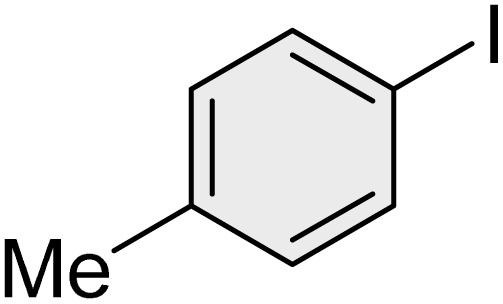	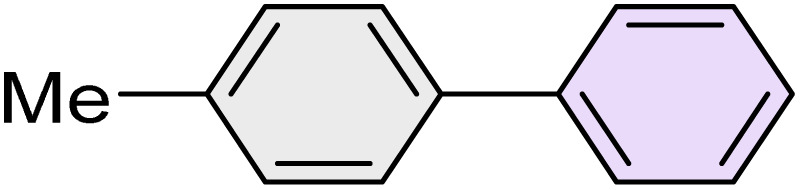	15	96	45–47	45–46 (ref. 67)
6	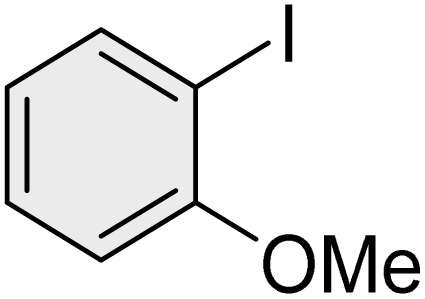	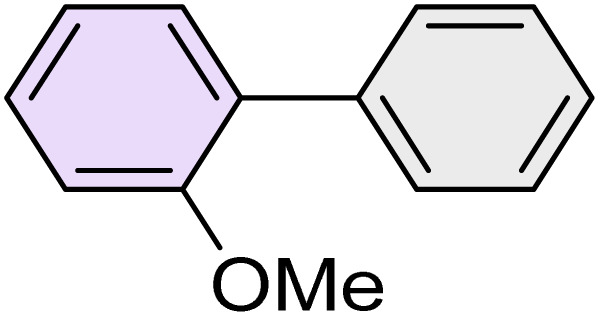	30	94	Oil	Oil (ref. 66)
7	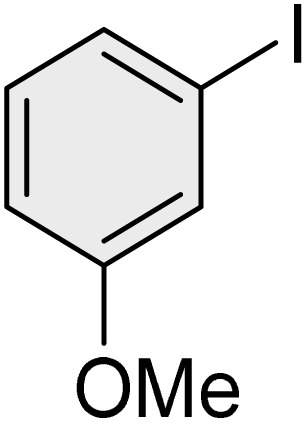	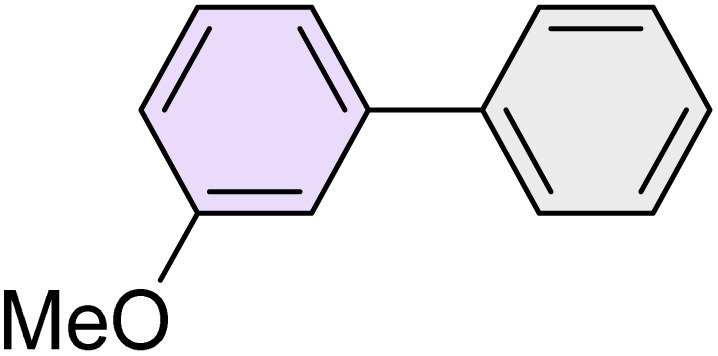	20	93	79–82	81–82 (ref. 68)
8	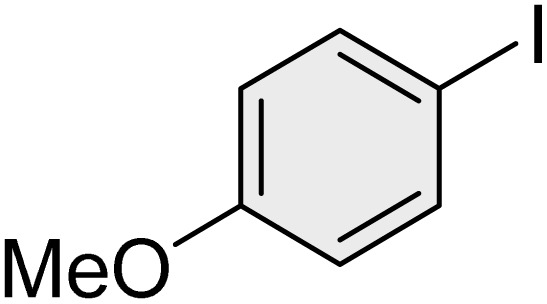	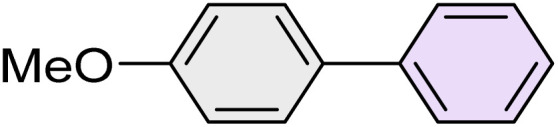	25	95	82–84	83–85 (ref. 67)
9	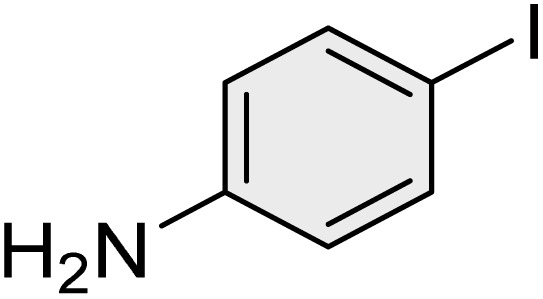	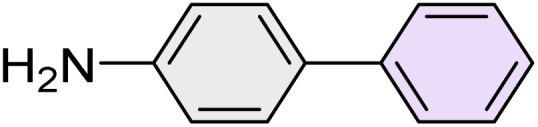	45	93	52–55	51–54 (ref. 69)
10	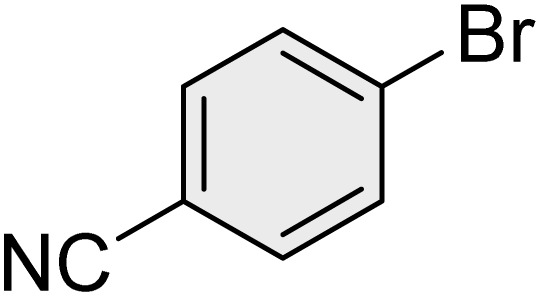	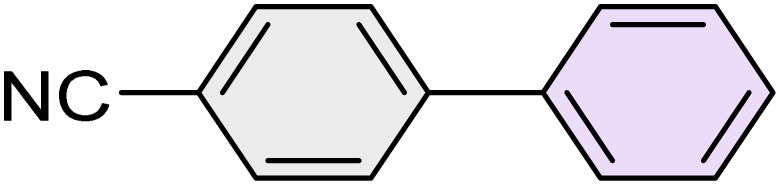	10	98	82–83	83 (ref. 70)
11	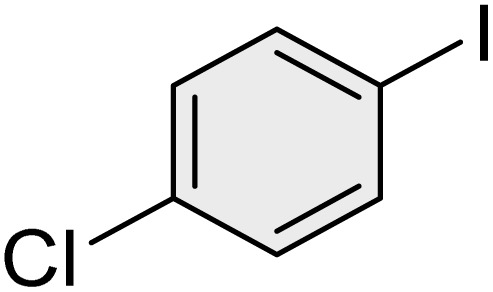	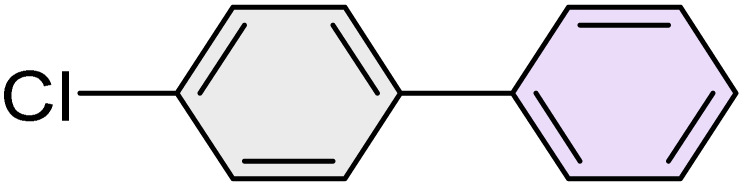	15	98	72–73	72–73 (ref. 56)

a Isolated yield.

b Conditions: aryl halides (1.0 mmol), phenylboronic acid (1.2 mmol), K_2_CO_3_ (3 mmol) and of [Fe_3_O_4_@UiO-66-NH(CH_2_CH_2_-2-picolylamine)_2_-Pd(0)] complex (1.3 mol%) in PEG-400 (2 mL) at 120 °C.

### 3.3 Reaction mechanism

**Scheme 2:**
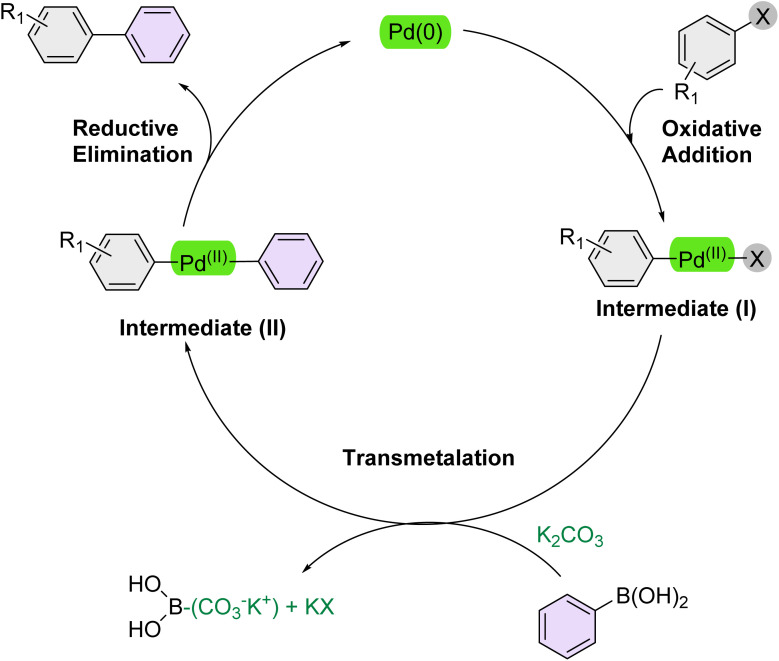
Plausible mechanism of the Suzuki reaction over the catalysis of [Fe_3_O_4_@UiO-66-NH(CH_2_CH_2_-2-picolylamine)_2_-Pd(0)] complex.

According to previous studies, Scheme 2 outlines a mechanistic pathway for the Suzuki reaction.^71^ The reaction commences with oxidative addition, where the Pd(0) catalyst is inserted into the carbon–halogen (C–X) bond of the organohalide, forming a new carbon–palladium (C–Pd(ii)–X) bond and cleaving the carbon–halogen bond. Subsequently, K_2_CO_3_ activates the arylboronic acid, enabling it to transfer its organic group to the Pd catalyst *via* transmetalation, which displaces a ligand. In the final step, reductive elimination expels both organic groups from the Pd(ii) complex, forming the desired carbon–carbon bond between the previously bound organic moieties and reducing the Pd(ii) catalyst back to Pd(0) which could be accelerated over the PEG-400 solvent.

### 3.4 Reusability study

**Fig. 10:**
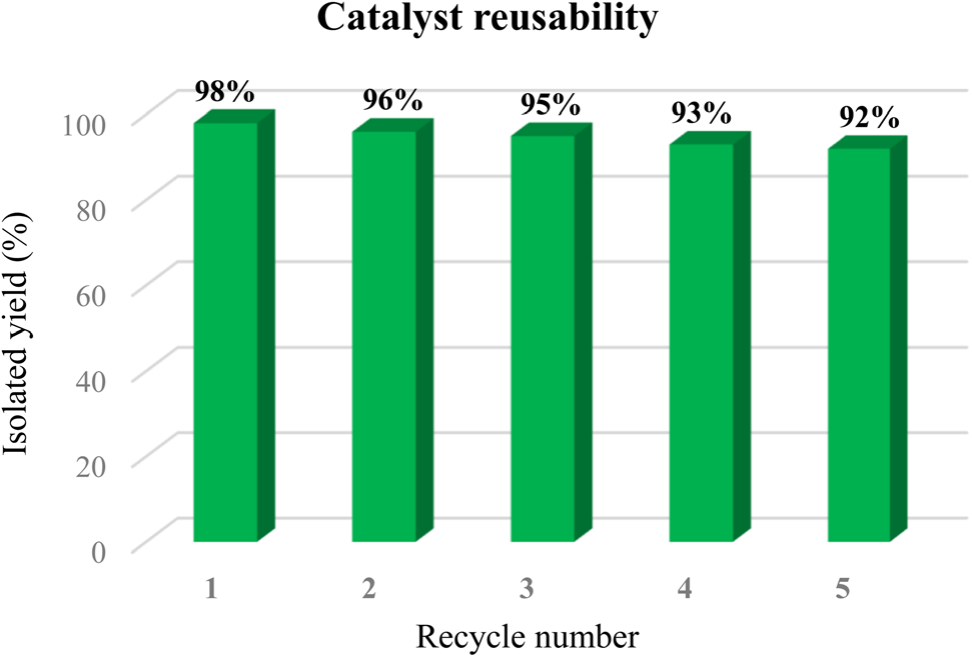
The reusability of [Fe_3_O_4_@UiO-66-NH(CH_2_CH_2_-2-picolylamine)_2_-Pd(0)].

To evaluate the catalyst's reusability, we performed several consecutive reaction cycles using the coupling of iodobenzene and phenylboronic acid as a model reaction under the established optimal conditions. After each run, the reaction mixture was diluted with ethyl acetate, and the catalyst was efficiently separated using a strong neodymium magnet. The isolated catalyst was then thoroughly washed with ethyl acetate and deionized water, followed by drying before its subsequent reuse. Remarkably, the catalyst maintained its high catalytic efficiency for at least five cycles, consistently achieving excellent product yields with negligible loss in efficiency despite an increased reaction time. These results, visually represented in Fig. 10, clearly demonstrate the catalyst's significant potential for effective recyclability.

**Fig. 11:**
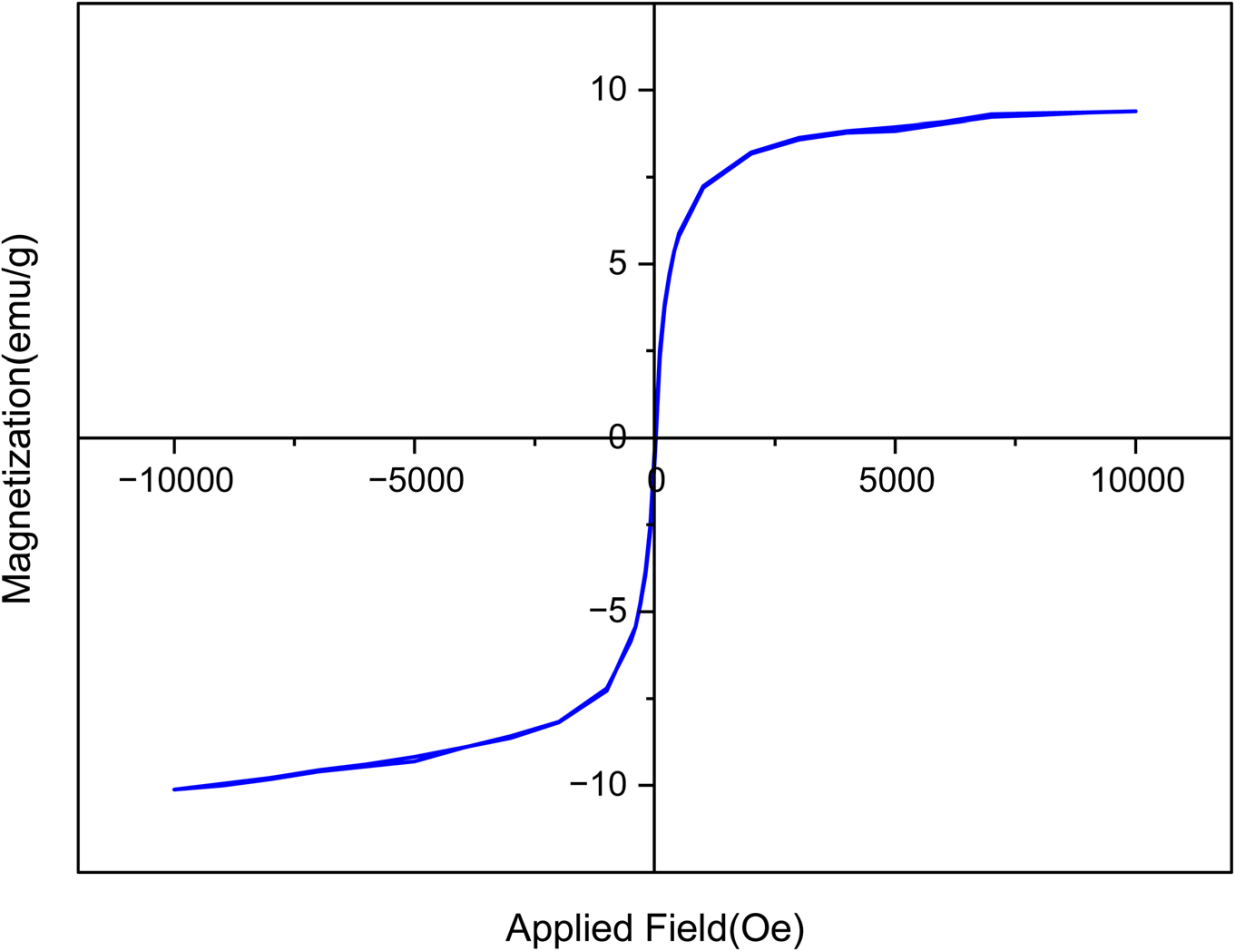
The VSM analysis of the recovered catalyst.

**Fig. 12:**
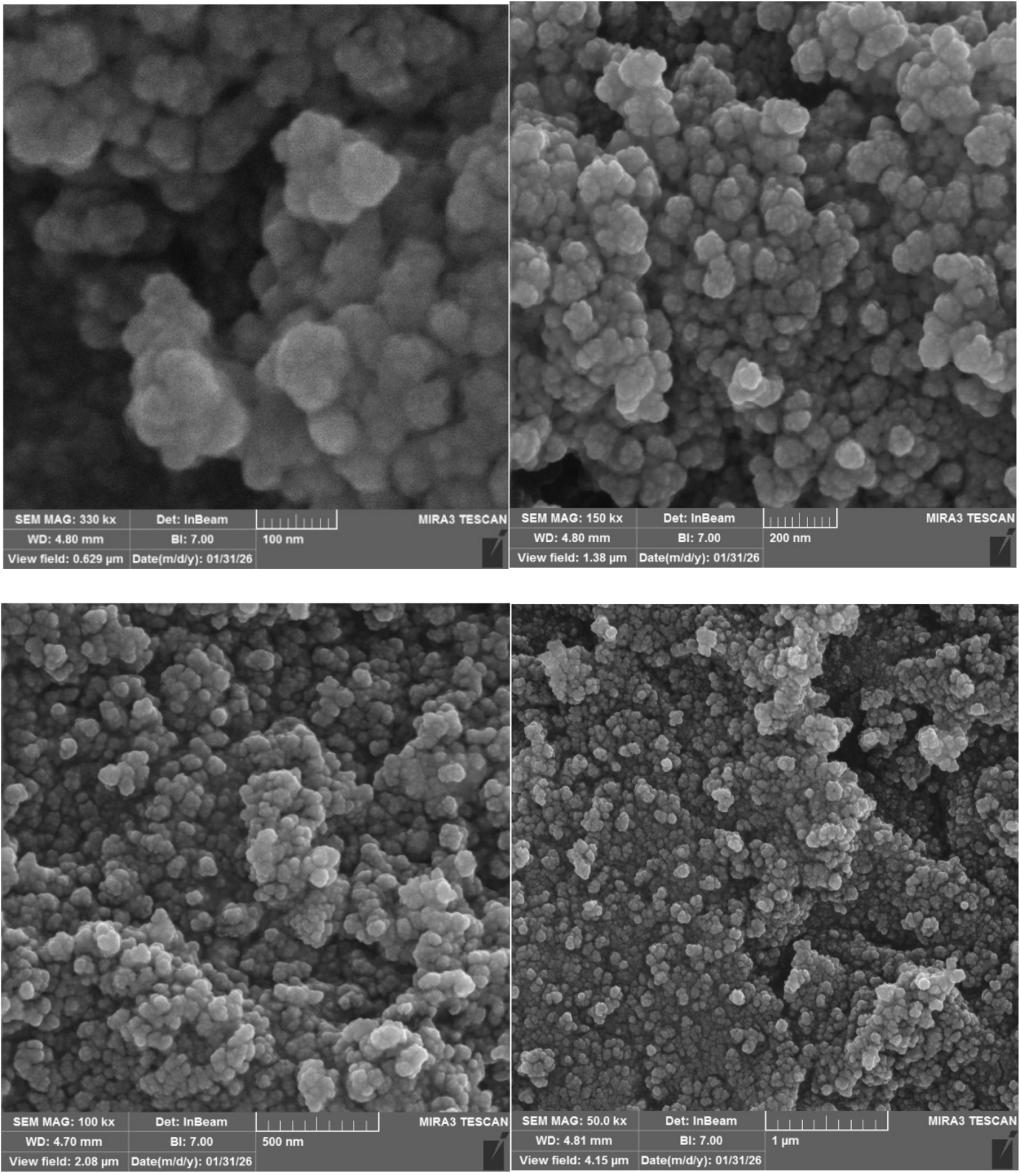
FE-SEM images of the recovered catalyst.

**Fig. 13:**
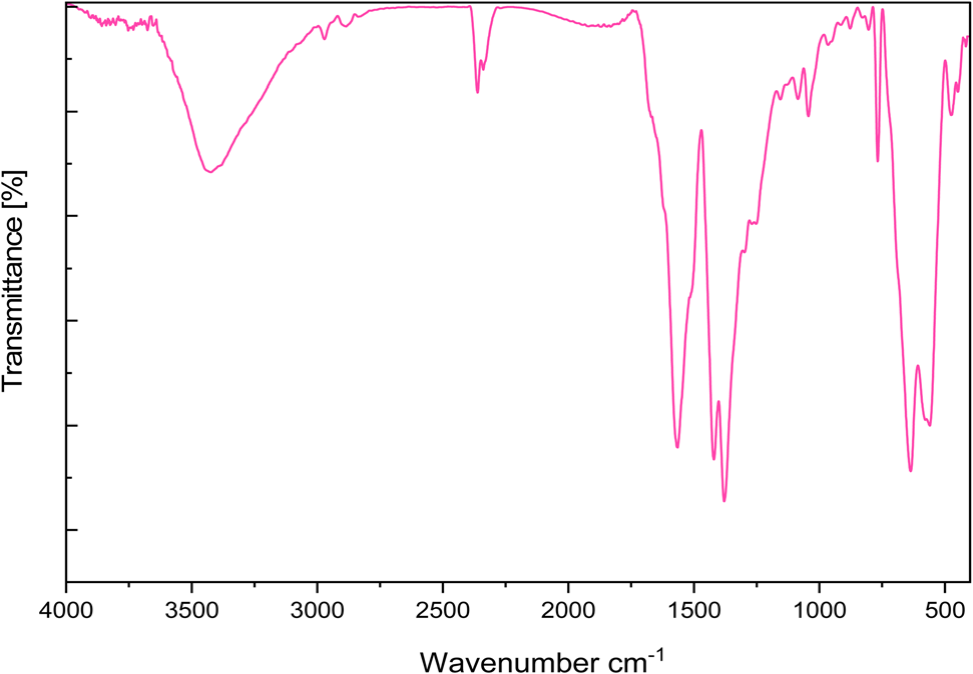
The FT-IR spectra of the recovered catalyst.

The magnetization curves in Fig. 11 display the magnetic stability of [Fe_3_O_4_@UiO-66-NH(CH_2_CH_2_-2-picolylamine)_2_-Pd(0)] nanocatalyst after the recovery process. According to the results, a slight decrease in the saturation magnetization (*M*_s_) was observed after several reaction cycles. However, the magnetic stability of the nanocatalyst was satisfactorily maintained for rapid separation from the reaction medium under the influence of an external magnetic field. Furthermore, morphological evaluation using FE-SEM images (Fig. 12) displayed that the [Fe_3_O_4_@UiO-66-NH(CH_2_CH_2_-2-picolylamine)_2_-Pd(0)] nanocatalyst after recovery still maintained its structural integrity, morphology and size distribution similar to the fresh sample. Ultimately, FT-IR analysis of the recovered catalyst exhibited no significant changes in the intensity, frequency, and shape of the absorption bands compared to the fresh catalyst (Fig. 13).

### 3.5 Hot-filtration test

To ascertain the nature of the catalyst in the reaction medium, a hot filtration test was performed using the above-mentioned model reaction. After 5 min of reaction, the catalyst was separated from the mixture using a strong neodymium magnet. At this point, a 67% yield of the desired product was obtained. The filtrate was then allowed to react for an additional 2 hours under the established optimal conditions. Subsequent reaction of the filtrate yielded no additional product beyond that obtained during the initial filtration. This observation confirms the completely heterogeneous nature of the catalyst.

### 3.6 Comparison

Finally, the performance of this novel catalytic method, using [Fe_3_O_4_@UiO-66-NH(CH_2_CH_2_-2-picolylamine)_2_-Pd(0)], was compared with other catalysts reported in the literature (Table 3). Many previously reported homogeneous and heterogeneous expensive transition metal catalysts for this transformation often required toxic ligands and solvents like DMF. The homogeneous systems also suffered from limited recovery and reusability. In contrast, our [Fe_3_O_4_@UiO-66-NH(CH_2_CH_2_-2-picolylamine)_2_-Pd(0)] catalyst demonstrates significant advantages in comparison to the best-known methodologies in literature. It achieves an average conversion of 98% in just 15 minutes, showcasing its exceptional efficiency. The primary benefits of our catalyst include its high selectivity, excellent reusability, and compatibility with greener reaction conditions, addressing key limitations of prior methods.

**Table 3:** Comparison of catalytic efficiency of [Fe_3_O_4_@UiO-66-NH(CH_2_CH_2_-2-picolylamine)_2_-Pd(0)] complex in synthesis of 1,1′-biphenyl

Entry	Catalyst (amount)	Solvent	Base	*T* (°C)	Time (min)	Yield (%)	Ref.
1	UiO-66-NH_2_@cyanuric chloride@guanidine/Pd-NPs (20 mg)	H_2_O	K_2_CO_3_	50	40	97	72
2	Pd@DCA-MCM(0.1 mol%)	H_2_O/EtOH	K_2_CO_3_	80	1440	94	73
3	MNP@PNHC-Pd(NP) (0.1 mol%)	H_2_O/EtOH	K_2_CO_3_	RT	30	74	74
4	Fe_3_O_4_@MON-Pd (0.2 mol%)	H_2_O/EtOH	K_2_CO_3_	25	30	99	75
5	Fe_3_O_4_@BP-MOF-PdCl_2_ (1.8 mol%)	EtOH	K_2_CO_3_	Reflux	25	98	76
6	[Fe_3_O_4_@UiO-66-NH(CH_2_CH_2_-2-picolylamine)_2_-Pd(0)] (1.3 mol%)	PEG-400	K_2_CO_3_	120	15	98	This work

## 4 Conclusion

In summary, a novel magnetically recoverable heterogeneous nanocatalyst, [Fe_3_O_4_@UiO-66-NH(CH_2_CH_2_-2-picolylamine)_2_-Pd(0)], was successfully engineered *via* a facile four-step strategy, notably bypassing the prerequisite for surface functionalization of the Fe_3_O_4_ core. This involved *in situ* growth of an amine-functionalized UiO-66 MOF shell directly onto bare Fe_3_O_4_ nanoparticles, followed by the immobilization of the picolylamine–palladium complex *via* a post-synthesis approach. Characterization confirmed the uniform growth of UiO-66-NH_2_ shell on magnetic nanoparticles with a particle size of 25 nm and a surface area of 235.36 m^2^ g^−1^. The structural integrity confirmed by XRD, TGA, ICP, EDX and FT-IR analyses, along with the appropriate distribution of palladium active centers, enabled maximum access of reactants to the active sites.

The as-synthesized nanocatalyst exhibited catalytic efficiency in Suzuki–Miyaura cross-coupling reactions, delivering high to excellent yields (up to 98%) under environmentally benign conditions within remarkably short reaction times. This superior performance is primarily attributed to the high dispersion and stabilization of Pd(0) species within the porous MOF framework, which effectively minimizes leaching and facilitates rapid mass transfer. Furthermore, the catalyst displayed outstanding structural stability and strong magnetic responsiveness (*M*_s_ = 10.68 emu per g), allowing for efficient magnetic recovery and consistent reusability for at least five consecutive cycles with negligible loss in activity. These collective attributes, combined with broad substrate scope and high chemoselectivity, establish this nanocomposite as a sustainable and highly potent platform for advanced organic transformations and green synthesis.

## Author contributions

P. Nasri conceptualized the study and executed all practical laboratory experiments, forming the basis of her PhD thesis. She further contributed to software utilization for data analysis and was responsible for the writing, review, and editing of the manuscript. M. Norouzi supervised the research project, contributed to its conceptualization, interpreted the data, and reviewed and edited the final manuscript.

## Conflicts of interest

The authors declare that they have no competing interests.

## Supplementary Material

NA-OLF-D5NA01048A-s001

## Data Availability

The data that support the findings of this study are available in the supplementary information (SI) of this article. Supplementary information: copies of ^1^H NMR (250 MHz), and ^13^C NMR (62.9 MHz) spectra for prepared products. See DOI: https://doi.org/10.1039/d5na01048a.
