# Supplementary material for: A novel palladium-2-picolylamine complex grafted on magnetic UiO-66-NH2 nanocomposites: as an efficient heterogeneous catalyst for fast Suzuki–Miyaura cross coupling
Source: Nanoscale Adv. 2026 Feb 23;8(6):2079–95. doi: 10.1039/d5na01048a (PMC12937538; doi:10.1039/d5na01048a)

Supplementary Information (SI) for Nanoscale Advances.
This journal is © The Royal Society of Chemistry 2026

A novel Palladium-2-picolylamine Complex Grafted on Magnetic UiO-66-NH_2 nanocomposites: As an efficient Heterogeneous catalyst for Fast Suzuki–Miyaura Cross-Coupling

Parastoo Nasri [a], Masoomeh Norouzi [a,*]

[a] *Department of Chemistry, Faculty of Science, Ilam University, P.O. Box 69315516, Ilam, Iran.*

[*] Correspondence to: (M. Norouzi), Ilam University, Ilam, Iran, E-mail: m.norozi@ilam.ac.ir

General

All solvents and chemical reagents were obtained from Merck Chemical Company and used without further purification. ^{1} H NMR and ^{13}C NMR spectra were recorded on a Bruker spectrometer in $CDCl_3$-d_6. Chemical shifts (δ) are presented in parts per million (ppm), while coupling constants (*J*) are expressed in Hertz (Hz). The following standard abbreviations were employed to describe the multiplicity of the signals: s = singlet, d = doublet, t = triplet, q f=or quartet, m = multiplet, and br = broad signals.

Spectral Data:

3-Methoxybiphenyl

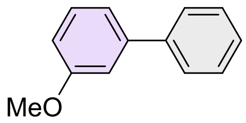

Yield = 93%; ^{1}H NMR (250 MHz, $CDCl_3$-d_6), δ (ppm): 7.73 (s, 2 H), 7.57–7.46 (m, 4 H), 7.33–7.29 (d, J = 8.4 Hz, 2H), 7.04 (s, 1H), 3.94 (s, 3 H). ^{13}C NMR (63 MHz, $CDCl_3$) δ: 160.09, 142.87, 141.21, 129.91, 128.89, 127.56, 127.33, 119.79, 113.02, 112.78, 55.33.

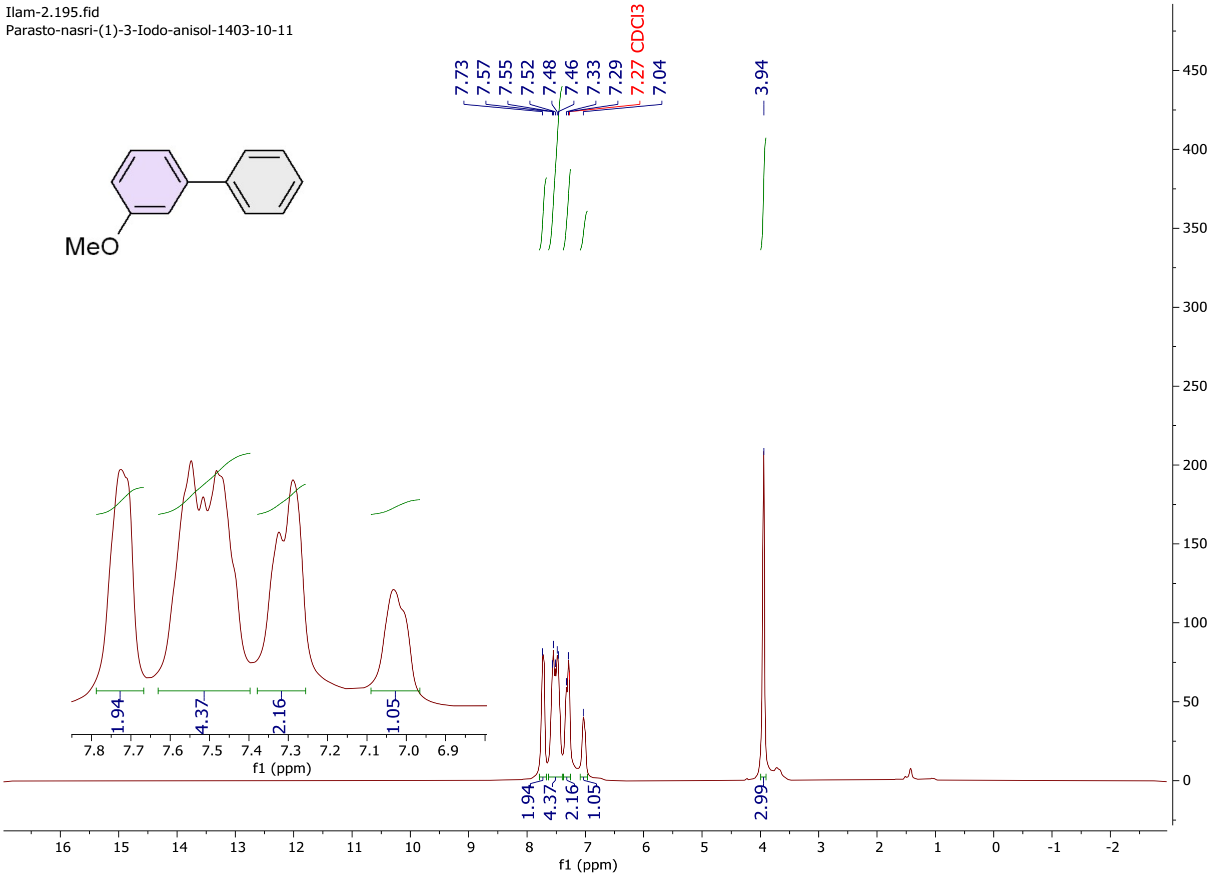

Figure S1. ^{1}HNMR spectrum of 3-Methoxybiphenyl

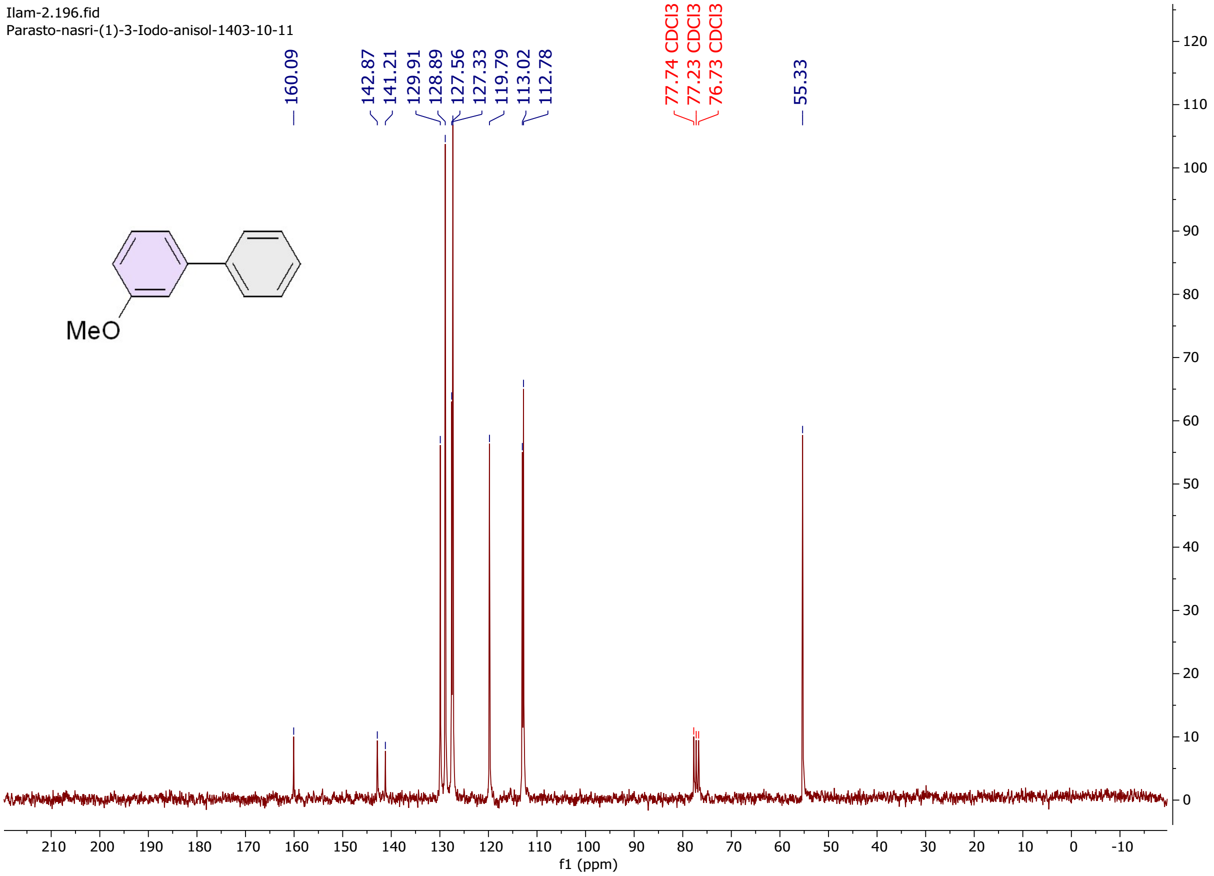

Figure S2. ^{13}CNMR spectrum of 3-Methoxybiphenyl

2-Methoxybiphenyl

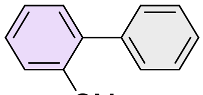

Yield = 94%; ^{1}H NMR (250 MHz, $CDCl_3$-d_6), δ (ppm): 7.63-7.7.60 (d, J = 7.8 Hz, 2H), 7.56 – 7.52-7.39 (m, 4H), 7.13-7.03 (q, J = 8.1 Hz, 2H), 3.86 (s, 3H). ^{13}C NMR (63 MHz, $CDCl_3$) δ 156.50, 138.60, 130.95, 130.75, 129.61, 128.68, 128.05, 126.98, 120.88, 111.25, 55.57.

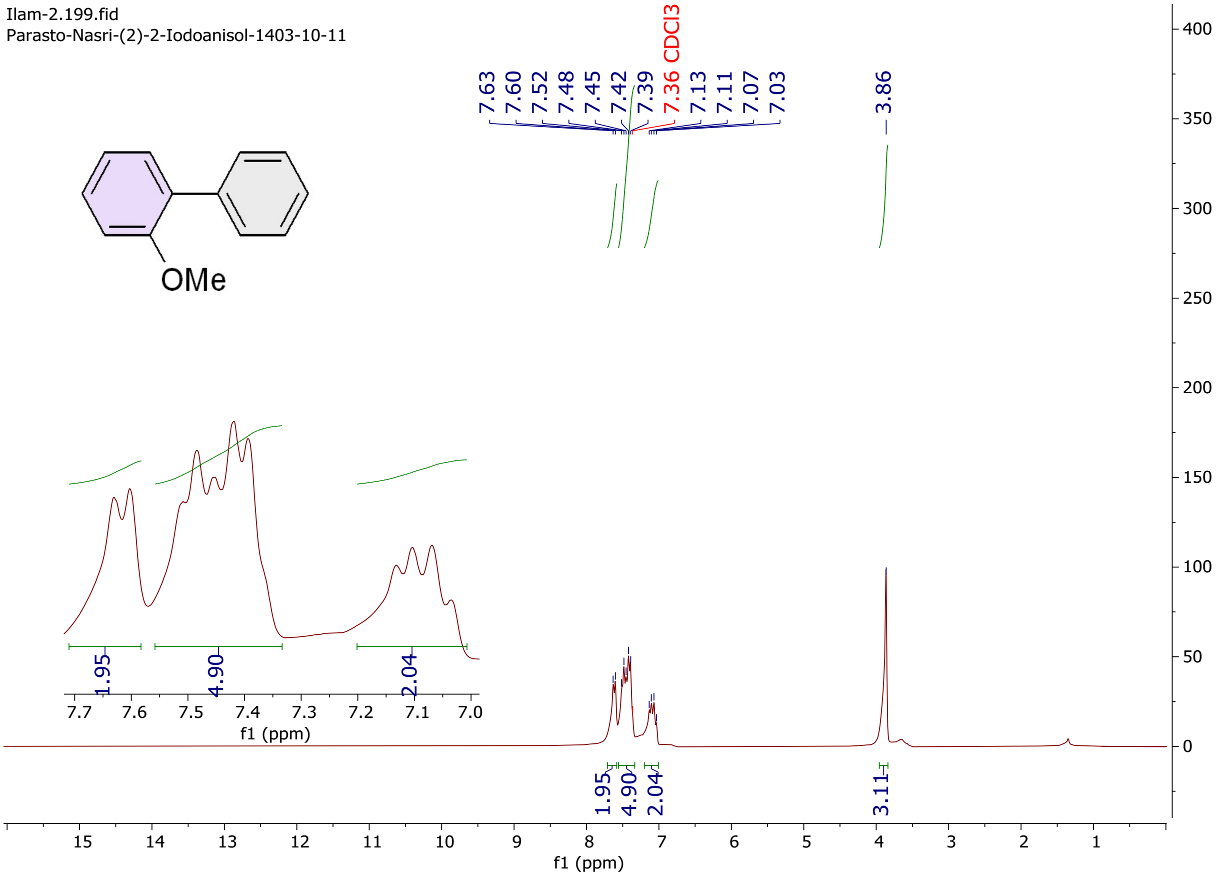

Figure S3. ^{1}HNMR spectrum of 2-Methoxybiphenyl

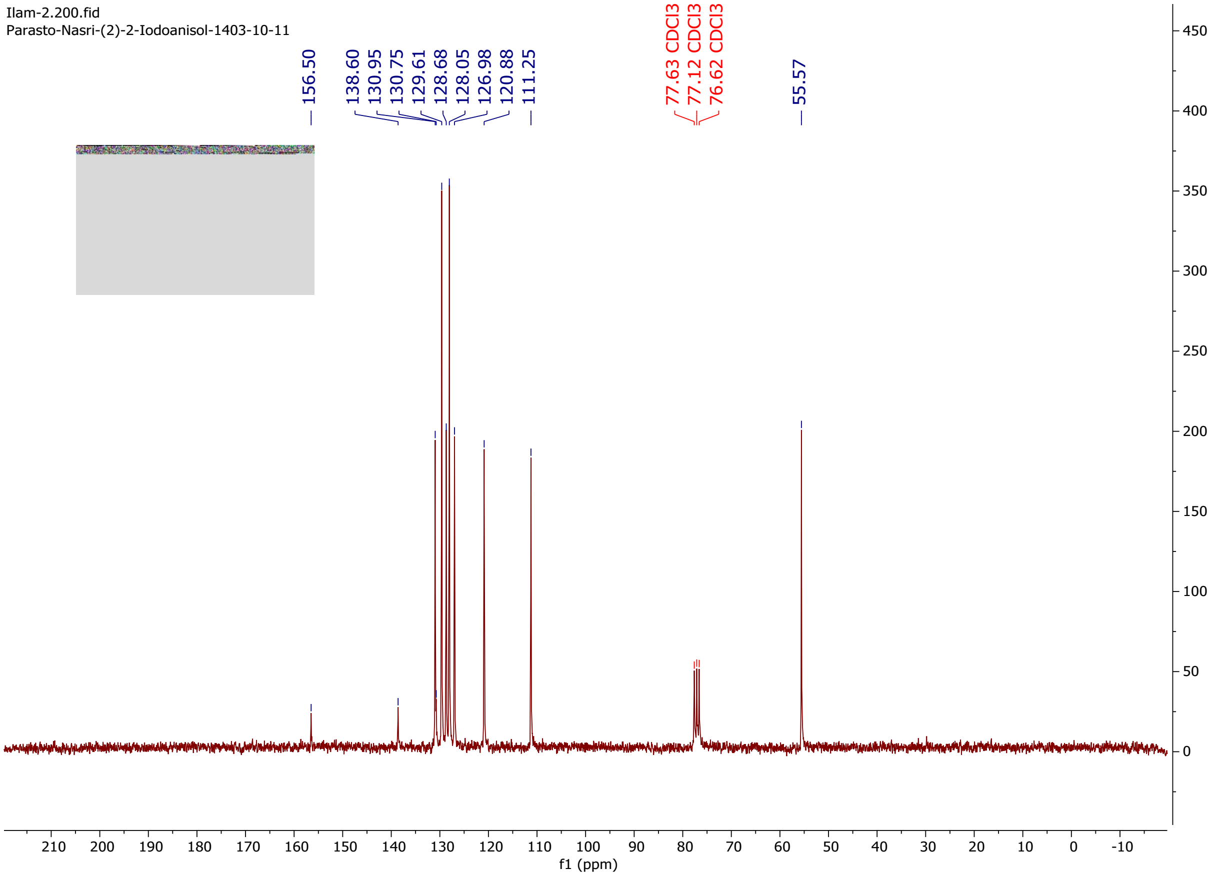

Figure S4. ^{13}CNMR spectrum of 2-Methoxybiphenyl

2-Methylbiphenyl

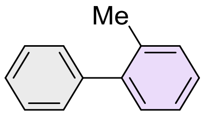

Yield = 94%; ^{1}H NMR (250 MHz, $CDCl_3$-d_6), δ (ppm): 7.43 – 7.25 (m, 9H), 2.29 (s, 3H). ^{13}C NMR (63 MHz, $CDCl_3$) δ 141.94, 135.35, 130.30, 129.79, 129.19, 128.06, 127.24, 126.76, 125.75, 20.48.

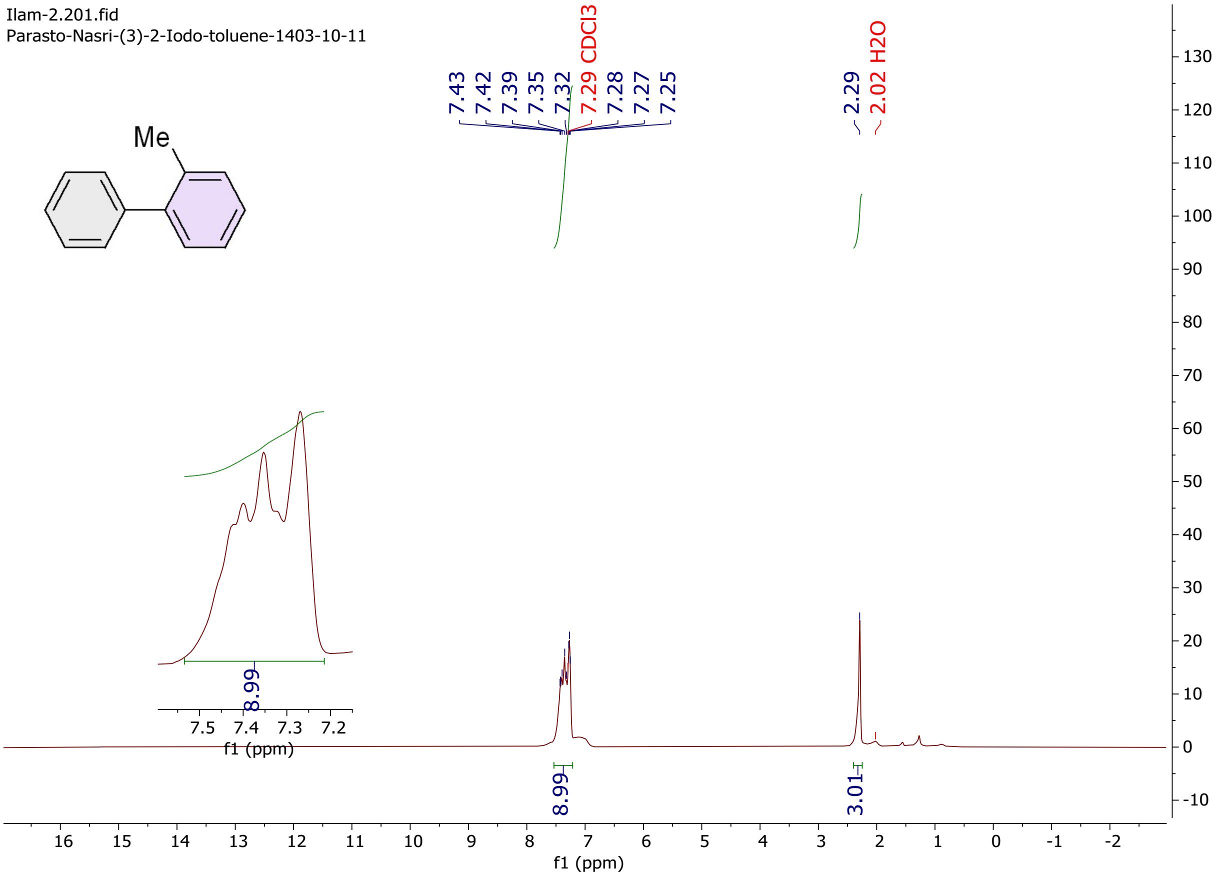

Figure S5. ^{1}HNMR spectrum of 2-Methylbiphenyl

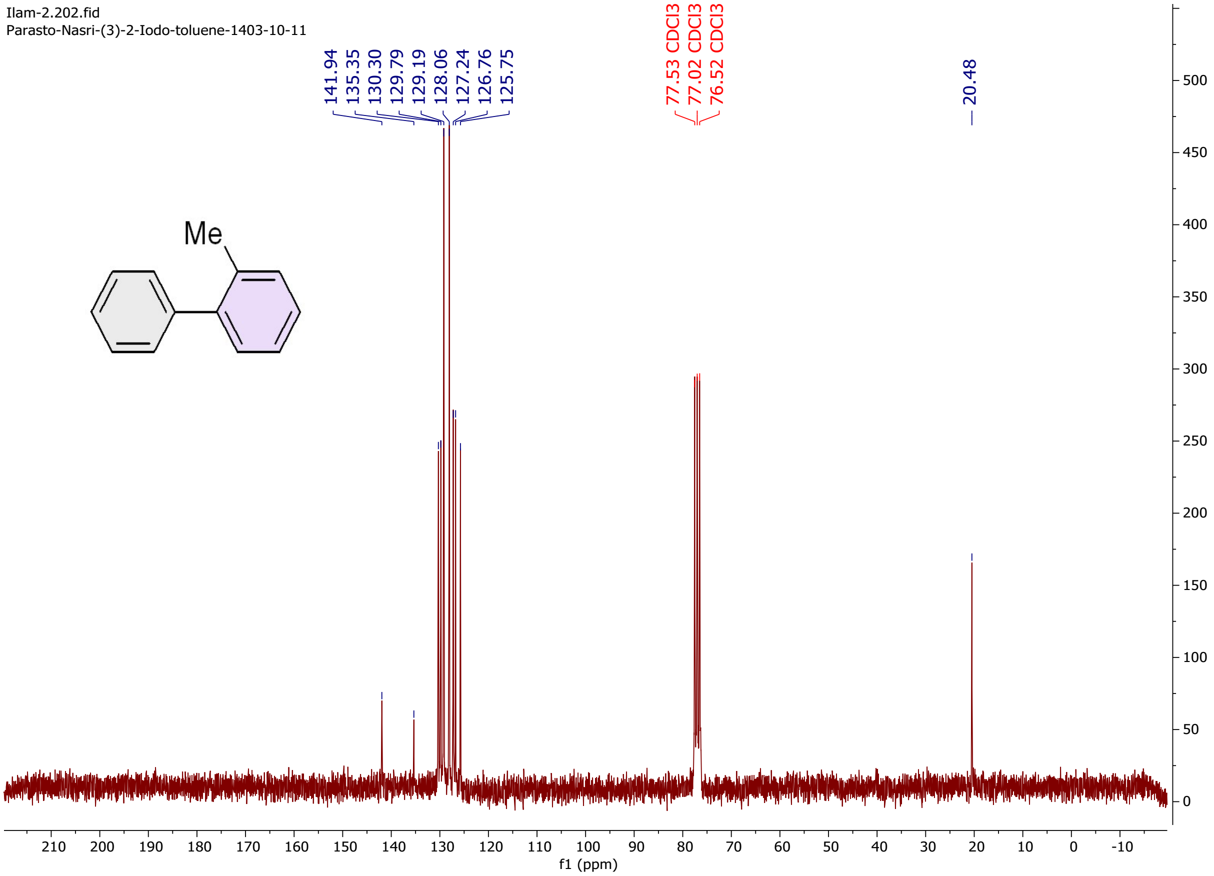

Figure S6. ^{13}CNMR spectrum of 2-Methylbiphenyl

4-Methylbiphenyl

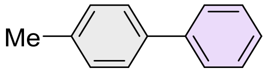

Yield = 96%; ^{1}H NMR (250 MHz, $CDCl_3$-d_6), δ (ppm): 7.70-7.62 (d, J = 8.3 Hz, 2H), 7.68-7.52 (d, J = 8.1 Hz, 2H), 7.49 – 7.36 (m, 5H), 2.49 (s, 3H). ^{13}C NMR (63 MHz, $CDCl_3$) δ 141.24, 138.44, 137.10, 129.59, 128.82, 127.10, 127.07, 21.21.

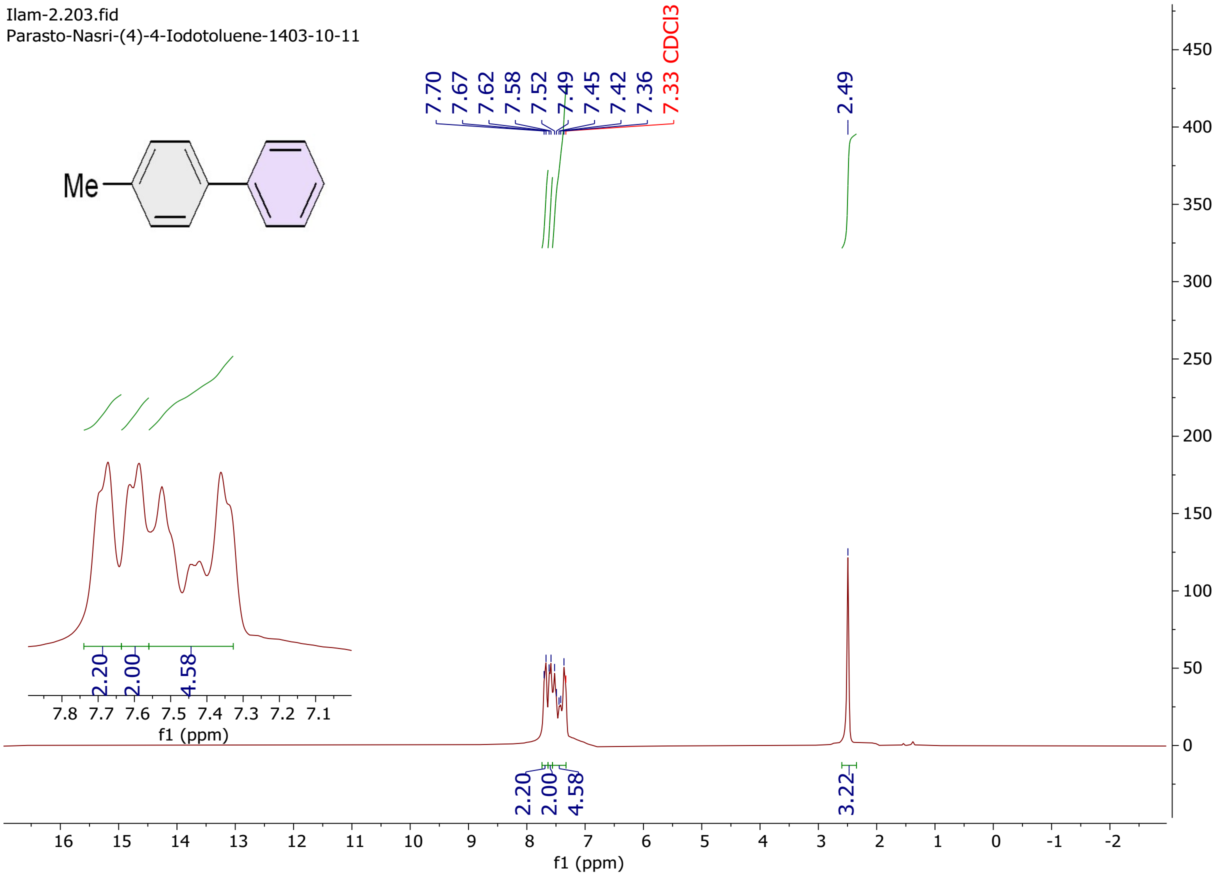

Figure S7. ^{1}HNMR spectrum of 4-Methylbiphenyl

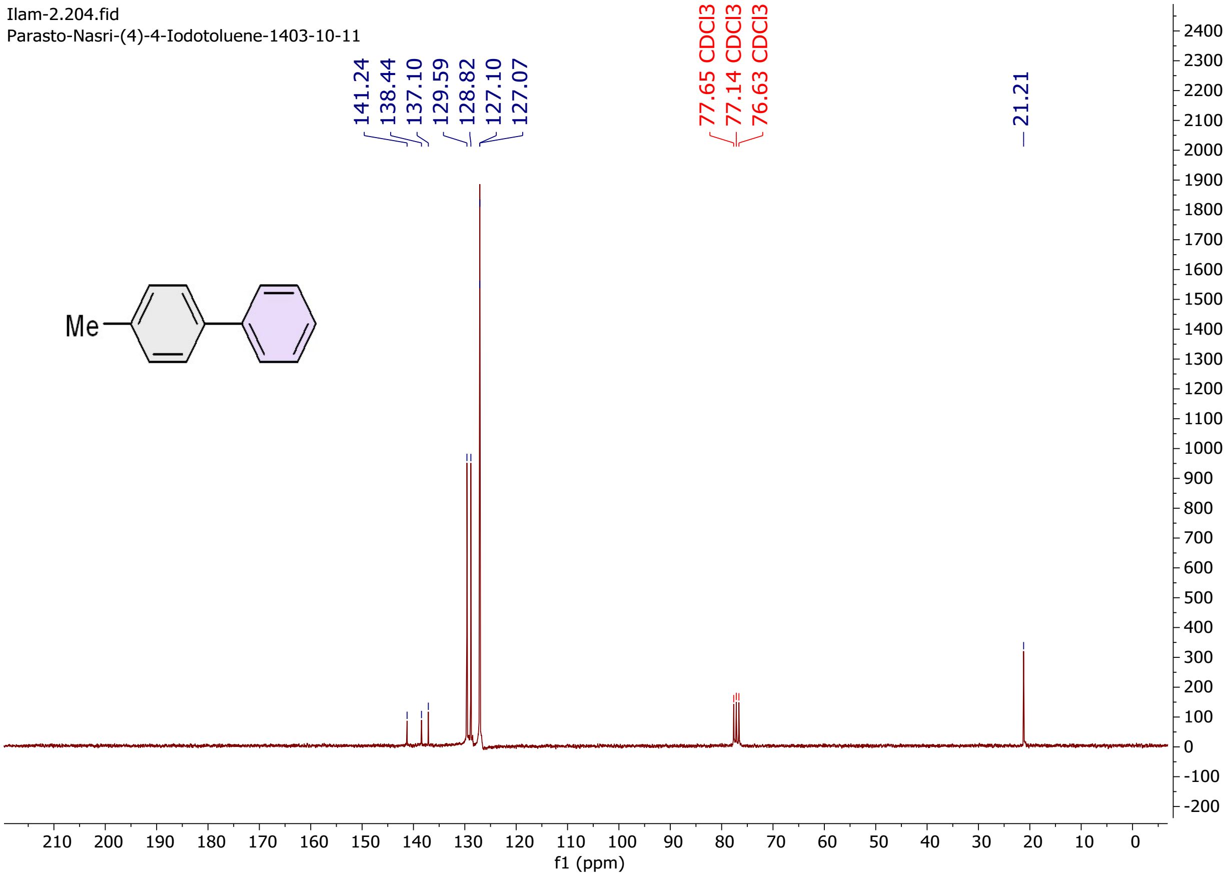

Figure S8. ^{13}CNMR spectrum of 4-Methylbiphenyl

4-Methoxybiphenyl

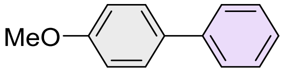

Yield = 95%; ^{1}H NMR (250 MHz, $CDCl_3$) δ 7.61 (t, J = 7.4 Hz, 4H), 7.48 (t, J = 7.6 Hz, 2H), 7.38 (d, J = 7.5 Hz, 1H), 7.04 (d, J = 8.6 Hz, 2H), 3.89 (s, 3H).. ^{13}C NMR (63 MHz, $CDCl_3$) δ 159.18, 140.86, 133.78, 128.79, 128.20, 126.79, 126.72, 114.24, 55.36.

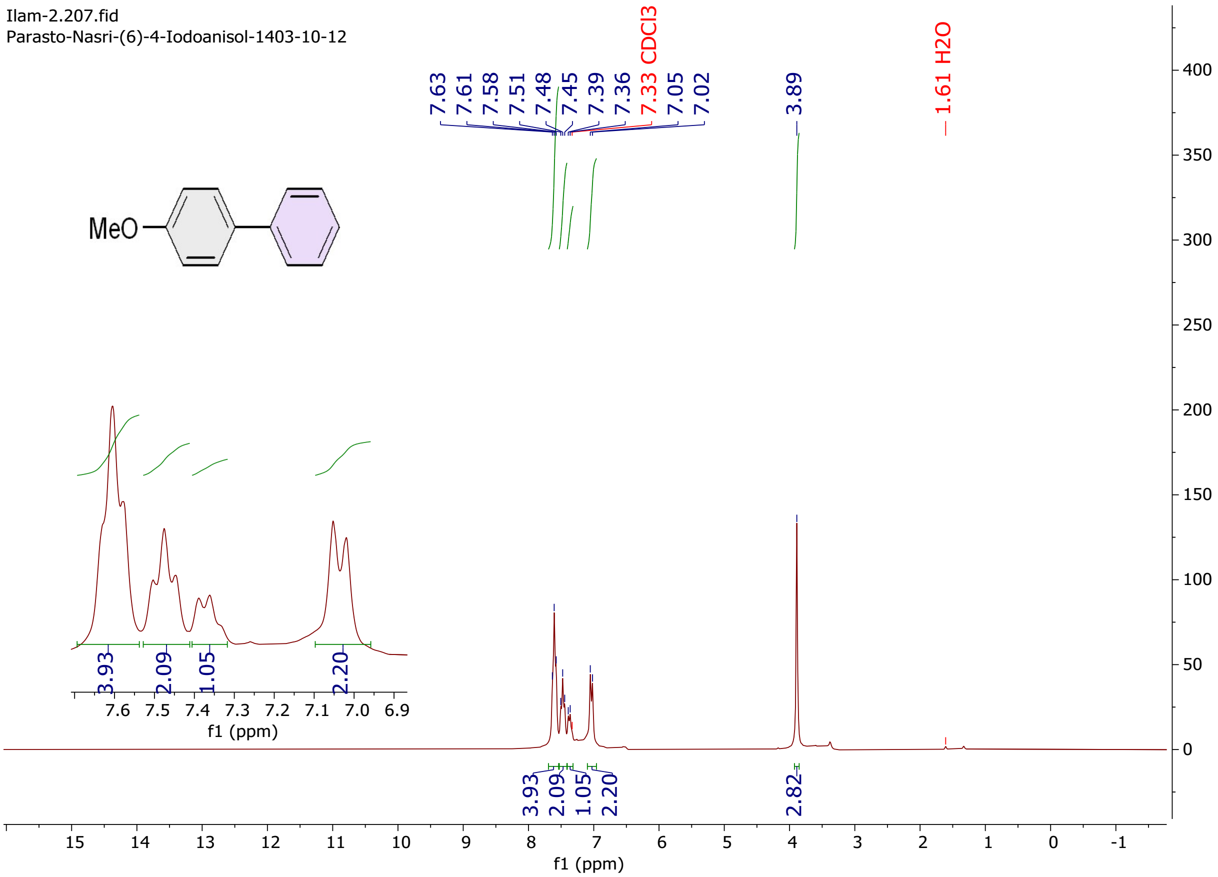

Figure S9. ^{1}HNMR spectrum of 4-Methoxybiphenyl

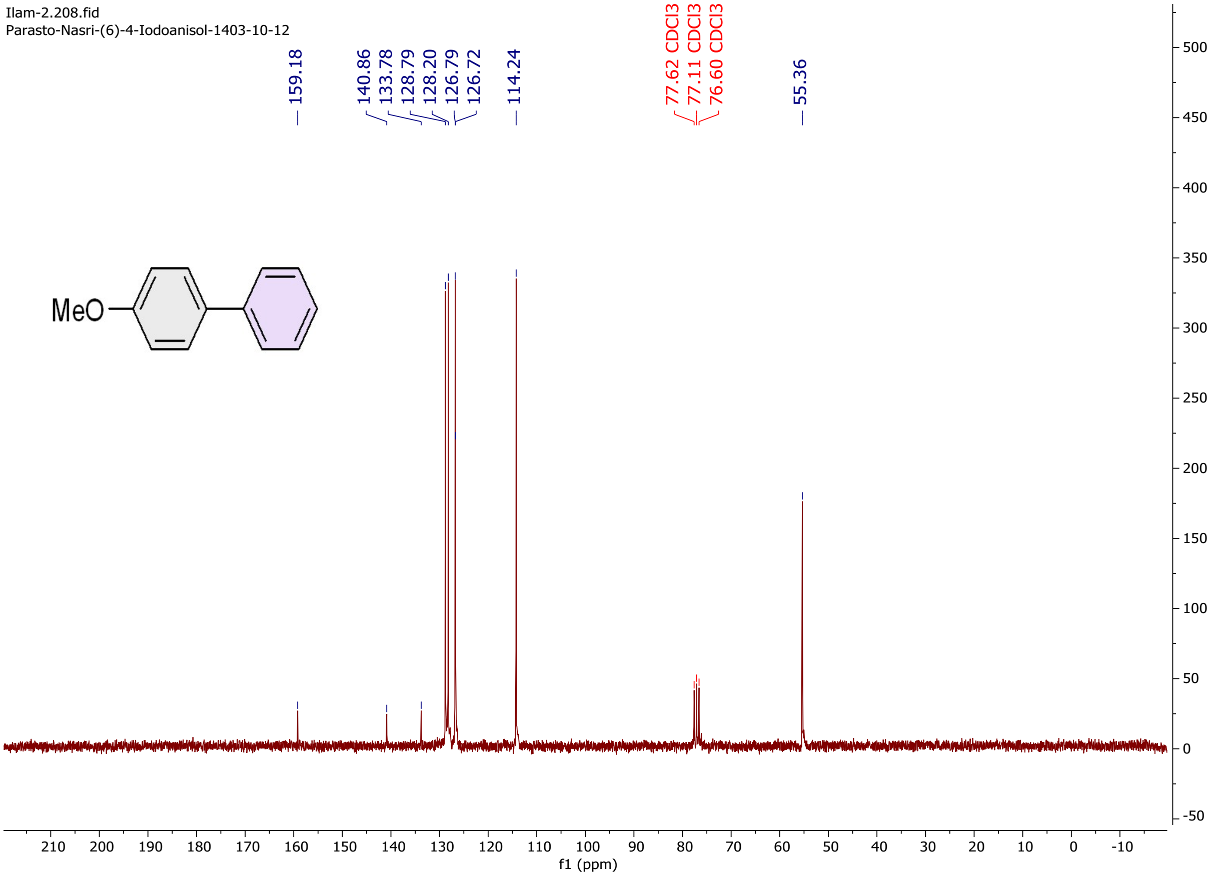

Figure S10. ^{13}CNMR spectrum of 4-Methoxybiphenyl

Biphenyl-4-carbonitrile:

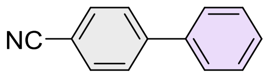

Yield = 98%, ^{1}H NMR (250 MHz, DMSO) δ 7.86 (d, J = 6.0 Hz, 4H), 7.71 (d, J = 7.8 Hz, 2H), 7.47 (d, J = 7.6 Hz, 3H). ^{13}C NMR (63 MHz, DMSO) δ 145.07, 138.69, 133.28, 129.61, 129.20, 128.00, 127.52, 119.32, 110.49.

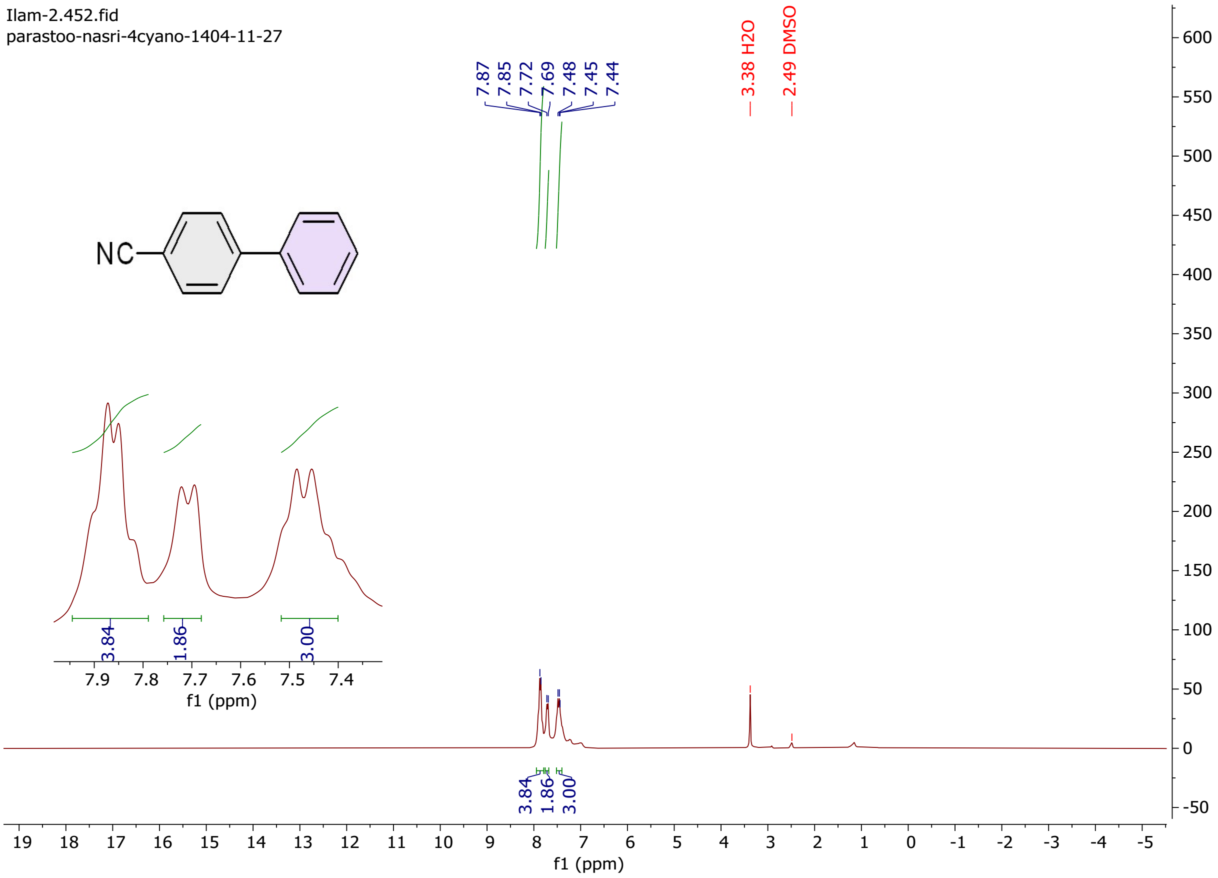

Figure S11. ^{1}HNMR spectrum of Biphenyl-4-carbonitrile

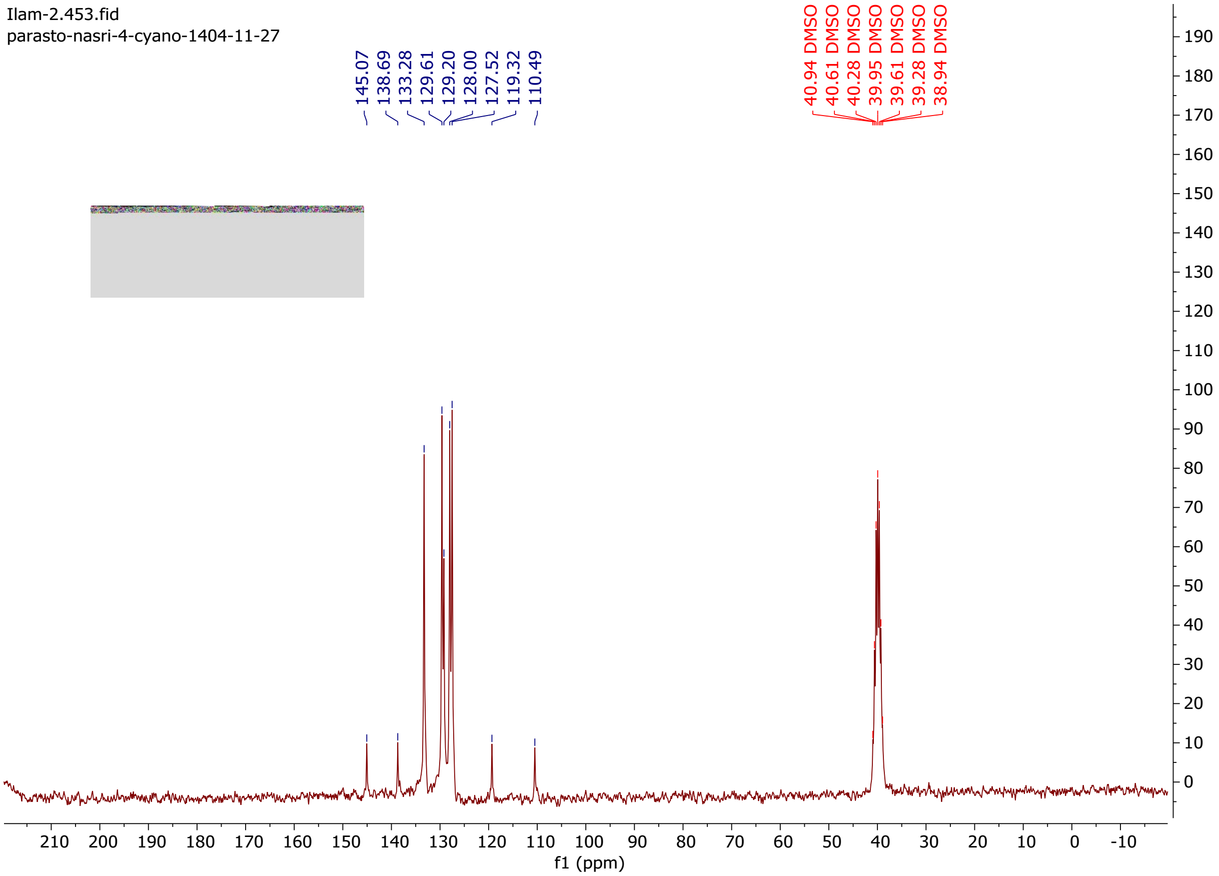

Figure S12. ^{13}CNMR spectrum of Biphenyl-4-carbonitrile

Biphenyl:

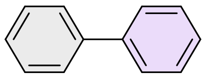

Yield = 98%, ^{1}H NMR (250 MHz, DMSO) δ 7.69 – 7.59 (m, 4H), 7.45 (t, *J* = 7.5 Hz, 4H), 7.36 (d, *J* = 7.4 Hz, 2H).

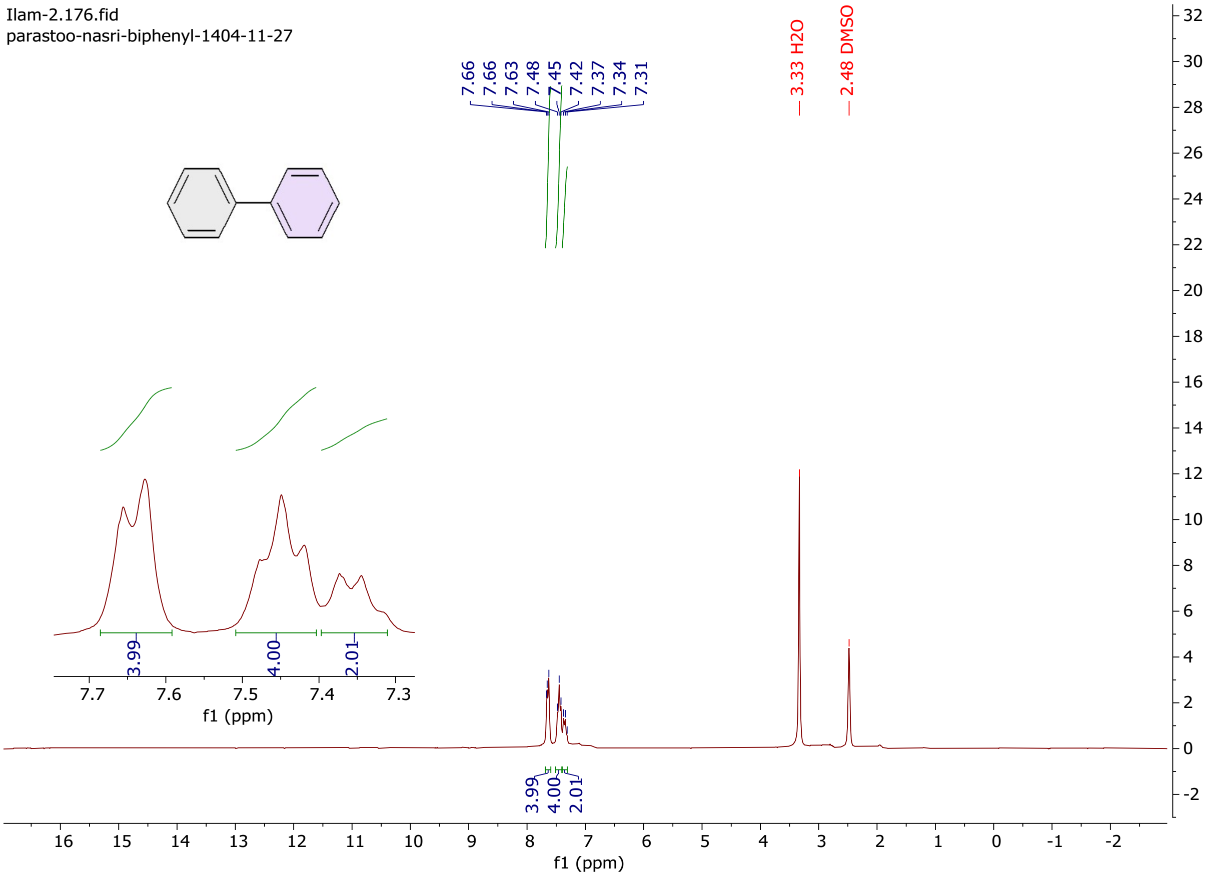

Supplement: NA-008-D5NA01048A-s001 [file NA-008-D5NA01048A-s001.pdf]
